# Lactylation Remodels Tumorigenesis, Immune Microenvironment, and Therapeutic Response

**DOI:** 10.3390/cimb48090926

**Published:** 2026-09-10

**Authors:** Yufei Liu, Yutong Zhou, Huiwen Xue, Ruiru Xu, Yujiao Liu

**Affiliations:** State Key Laboratory of Separation Membranes and Membrane Processes, School of Chemistry, Tiangong University, Tianjin 300387, China; 2315210212@tiangong.edu.cn (Y.L.);

**Keywords:** lactylation, tumor metabolic reprogramming, tumor immune microenvironment, targeted therapy, lactate, post-translational modification

## Abstract

Lysine lactylation (Kla) is a lactate-driven post-translational modification that covalently links lactyl groups to lysine residues, directly coupling cellular metabolic states to gene expression regulation and protein functional remodeling. Since its first report in 2019, extensive studies have confirmed that lactylation is broadly present on histones and thousands of non-histone substrates. In the context of tumor biology, lactylation reinforces glycolysis through positive feedback loops, suppresses oxidative phosphorylation, remodels lipid and glutamine metabolism, and exerts regulatory functions in autophagy, pyroptosis, ferroptosis, and apoptosis, thereby comprehensively participating in tumor cell proliferation, metabolic adaptation, cell death resistance, and invasion and metastasis. Within the tumor microenvironment, lactylation constructs an immune evasion barrier by upregulating immune checkpoints, including programmed death-ligand 1 (PD-L1), driving tumor-associated macrophage polarization toward the M2 phenotype, inducing CD8^+^ T cell exhaustion, and enhancing regulatory T cell suppressive function. Strategies targeting lactate production (lactate dehydrogenase A (LDHA) inhibitors), lactate transport (monocarboxylate transporter (MCT) inhibitors), and the lactylation enzymatic machinery (p300/CBP inhibitors, histone deacetylase (HDAC) inhibitors) have shown promising results in preclinical models, and a limited number of agents, including the MCT1 inhibitor AZD3965 and the p300/CBP inhibitor CCS1477, have entered early-phase clinical trials primarily for safety and tolerability assessment. This review systematically summarizes the molecular mechanisms and enzymatic basis of lactylation, as well as its regulatory functions in core cancer hallmarks and the immune microenvironment, evaluates the translational prospects of targeting the lactate–lactylation axis, and discusses the key scientific questions and future research directions currently facing the field.

## 1. Introduction

Tumor cells enhance glycolysis to sustain the biosynthetic precursors required for rapid proliferation, preferentially converting glucose into lactate even under aerobic conditions, a phenomenon termed the Warburg effect. For a long time, lactate was regarded as a terminal metabolic waste product of glycolysis, primarily exported extracellularly via monocarboxylate transporters (MCTs) to maintain intracellular pH homeostasis. However, research over the past two decades has upended this notion. Isotopic tracer techniques have revealed that lactate is not only a significant energy substrate but can also directly enter the tricarboxylic acid (TCA) cycle as a preferred carbon source through lactate shuttling, with its metabolic contribution even exceeding that of glucose [1]. High lactate accumulation in solid tumors correlates with poor prognosis, increased metastasis, and immunotherapy resistance, suggesting that lactate is a critical driver rather than an inert byproduct [2,3]. Moreover, lactate functions as a signaling molecule by binding to G protein-coupled receptors such as GPR81, activating pro-angiogenic and immunosuppressive cascades [4,5,6].

In 2019, the team of Yingming Zhao at the University of Chicago reported, for the first time in *Nature*, a novel acylation modification, histone lysine L-lactylation (Kla), unveiling the chemical mechanism by which lactate directly participates in epigenetic regulation [7]. This discovery has since expanded from histones to thousands of non-histone proteins, revealing that lactylation, like phosphorylation and acetylation, is highly dynamic and reversible. A growing list of writers and erasers has been identified, and recent studies have even uncovered lactyl-CoA-independent lactylation pathways. This reversibility enables cells to rapidly respond to fluctuating lactate levels and positions lactylation as a promising therapeutic node [8,9,10,11].

Although research on lactylation modification remains in a phase of rapid development, accumulating evidence indicates that it exerts multiple regulatory functions in tumor initiation and malignant progression. At the cellular level, lactylation drives glycolysis enhancement through a positive feedback loop. It reinforces metabolic reprogramming and cell survival pathways; within the tumor microenvironment, it orchestrates immune evasion by modulating multiple immune cell populations and checkpoint molecules [12,13]. Furthermore, lactylation of DNA repair proteins has been linked to chemoradiotherapy resistance. Collectively, these findings position lactylation as a pro-tumor network hub that integrates metabolic reprogramming, epigenetic regulation, DNA damage response, and immune evasion.

Given the multifaceted roles of lactylation in tumor biology, therapeutic strategies targeting the lactate–lactylation axis have garnered considerable attention. It should be emphasized at the outset, however, that the vast majority of evidence supporting these strategies remains preclinical, and clinical data are scarce. These strategies can be categorized into three interconnected levels. The first level targets lactate production and transport to reduce substrate supply for lactylation. The second level directly targets the lactylation enzymatic machinery, blocking lactate transport. The third level focuses on combination strategies of lactylation-targeting agents with immunotherapy. Preclinical studies have reported antitumor activity for each of these categories, and selected agents have advanced to early-phase clinical trials. Multiple preclinical studies have also suggested that lactate dehydrogenase A (LDHA) inhibitors, MCT inhibitors, and HDAC inhibitors can synergize with anti-PD-1/PD-L1 antibodies, with mechanisms converging on alleviating lactate-mediated immunosuppression and restoring T cell function. However, clinical translation still faces obstacles, including the lack of standardized lactylation quantification methods, cross-interference from the acetylation network, and the functional divergence of lactylation between tumor cells and immune cells. These challenges urgently require optimization of dosing regimens and patient stratification strategies.

This review aims to systematically delineate the metabolic origins and enzymatic regulatory basis of lactylation, dissect its functions in core cancer hallmarks including proliferation, metabolic reprogramming, cell death resistance, and metastasis, and critically elucidate the multi-layered mechanisms by which lactylation reshapes the tumor immune microenvironment. Furthermore, we provide a critical assessment of the preclinical evidence, clinical progress, and future translational challenges of lactylation-targeting strategies. Through this comprehensive synthesis, we seek to demonstrate that lactylation not only constitutes a critical molecular bridge connecting metabolism and epigenetics but also holds potential as a novel actionable target to overcome current bottlenecks in cancer immunotherapy.

## 2. Molecular Mechanisms and Regulatory Networks of Lactylation

As a novel lysine acylation modification derived from lactate, protein lactylation has been recognized in recent years as an important post-translational modification mechanism that bridges cellular metabolic states with signal transduction and gene expression regulation. To comprehend the functional logic of lactylation under physiological and pathological conditions, we need to understand the metabolic sources of lactate and its multi-source supply characteristics within the microenvironment, the non-enzymatic and enzymatic generation pathways of lactyl-CoA, the discovery of novel lactyltransferases, the substrate selectivity and functional division of labor among delactylase families, the diversity of histone versus non-histone substrates and their differential functional outputs, as well as how the dynamic regulation of lactylation senses intracellular metabolic fluctuations and engages in competitive crosstalk with other acylation modifications.

### 2.1. Metabolic Sources of Lactate

The massive accumulation of lactate within tumor cells is a direct metabolic consequence of the Warburg effect, a phenomenon also observed in activated immune cells and inflamed tissues. Biochemically, pyruvate derived from glycolysis is reduced to lactate by lactate dehydrogenase (LDH), with concomitant regeneration of NAD^+^. Although this pathway yields relatively low energetic efficiency, its rapid reaction kinetics and independence from mitochondrial integrity provide a critical metabolic foundation for the rapid proliferation of tumor cells [2]. Glutaminolysis constitutes another important source of lactate, with its contribution becoming particularly significant under glucose-limited conditions. Glutamine is converted to glutamate by glutaminase, which is then transaminated to α-ketoglutarate and enters the TCA cycle. Its metabolic intermediates derived from this pathway, including oxaloacetate, malate, and pyruvate, are ultimately converted to lactate by LDHA. This pathway simultaneously provides NADPH to support fatty acid synthesis and antioxidant defense. In addition to endogenous production, circulating lactate may also derive from gut microbial fermentation products that enter the bloodstream. Although serum lactate concentrations (approximately 100–200 μM) are far lower than those within the tumor microenvironment (10–30 mM), isotope tracing studies have confirmed that circulating lactate can be taken up by peripheral tissues and enter the TCA cycle, with its contribution to the TCA cycle in tumors even exceeding that of glucose.

**Figure 1:**
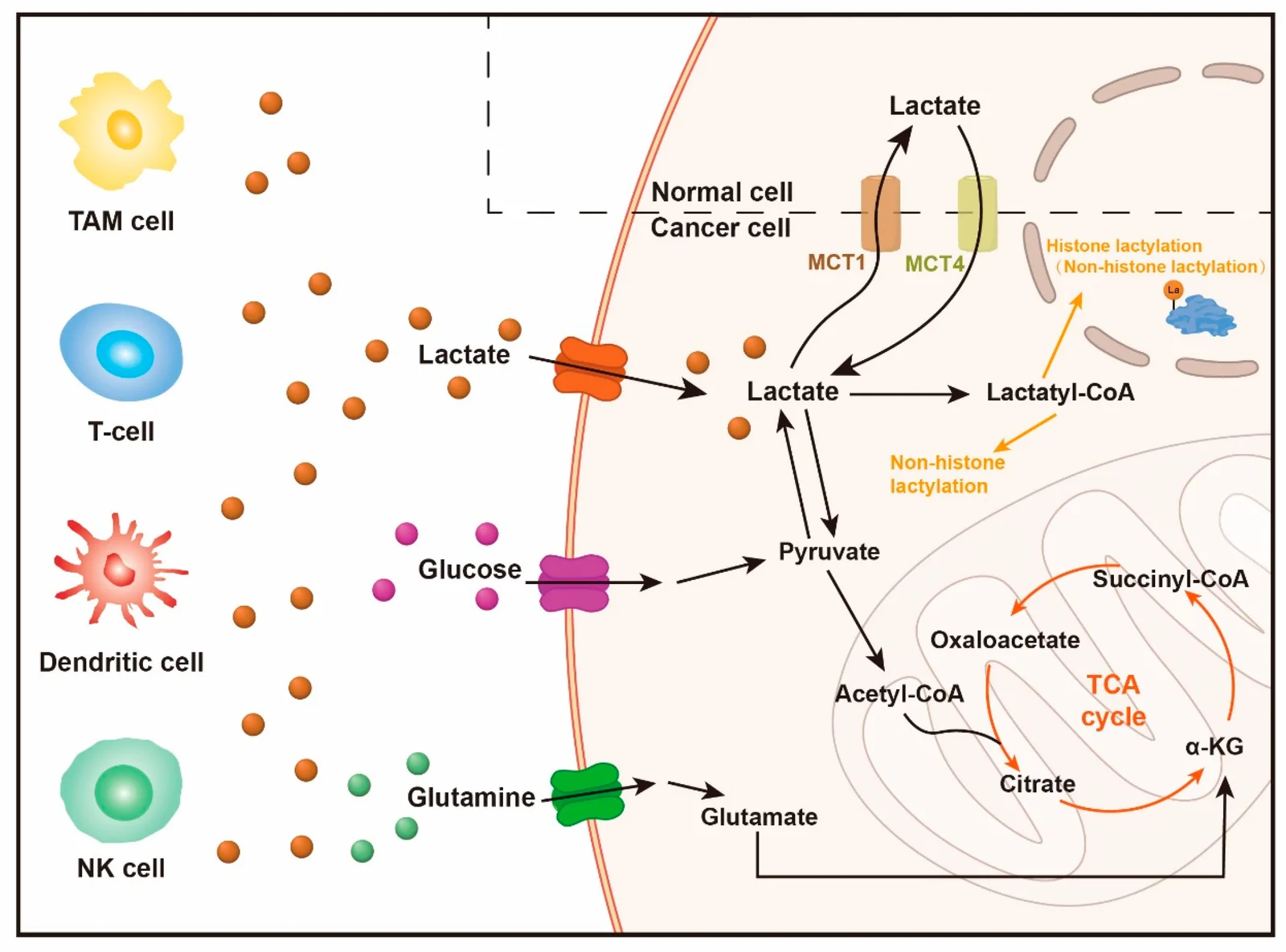
Generation of lactylation and its substrates.

Figure 1 provides an overview of lactate metabolism and the lactylation process (Figure 1). Within the tumor microenvironment, glucose metabolism, glutaminolysis, and circulating/local lactate uptake form a dynamically integrated network through the differential expression of MCT1 and MCT4, enabling both the uptake of exogenous lactate for oxidative metabolism and the export of endogenous lactate to maintain pH homeostasis [14]. This multi-source supply and bidirectional transport position lactate as a critical hub connecting metabolic reprogramming with microenvironmental remodeling and provide the concentration-dependent substrate foundation for subsequent lactylation.

### 2.2. Generation of Lactyl-CoA and Lactyltransferases

The occurrence of lactylation depends on the conversion of lactate to an activated donor molecule and the subsequent transfer of the lactyl group to substrate proteins. For a long time, it has been generally accepted that lactyl-CoA serves as the direct donor of lactylation, and its synthesis and utilization constitute the core biochemical basis of this modification. Recent breakthrough studies have not only identified the key enzymatic systems responsible for lactyl-CoA synthesis but have also uncovered alternative pathways that bypass lactyl-CoA, fundamentally expanding our understanding of the catalytic mechanisms underlying lactylation.

As the direct donor for lactylation, lactyl-CoA exhibits intracellular concentrations significantly lower than those of major acyl-CoA molecules such as acetyl-CoA. Quantitative analyses have shown that lactyl-CoA levels in mammalian cells and tissues range from 1/350 to 1/20 of those of acetyl-CoA [15,16]. This substantial concentration disparity suggests that the synthesis of lactyl-CoA may be subject to stringent regulation and that not all lactylation events rely on the lactyl-CoA pathway.

#### 2.2.1. Generation of Lactyl-CoA

Lactyl-CoA generation involves two independent enzymatic pathways. One is the ACSS2 pathway. Zhu et al. (2025) demonstrated that epidermal growth factor receptor (EGFR) activation induces extracellular regulated protein kinases (ERK) signaling-mediated phosphorylation of acyl-CoA synthetase short-chain family member 2 (ACSS2) at S267, promoting its nuclear translocation and formation of a complex with KAT2A. Within this complex, ACSS2 functions as a lactyl-CoA synthetase to directly convert lactate to lactyl-CoA, while KAT2A utilizes lactyl-CoA to catalyze histone H3 lactylation [17].

The second pathway is mediated by GTP-succinyl-CoA synthetase (GTPSCS). Liu et al. (2025) found that GTPSCS contains a nuclear localization sequence (192-KKGR-195), enabling its translocation to the nucleus under specific conditions, where it directly catalyzes lactyl-CoA generation and cooperates with p300 to drive H3K18la [18]. These two type-specific pathways operate in parallel, constituting enzymatic redundancy in lactyl-CoA supply, as shown in Table 1.

#### 2.2.2. The Lactyltransferases

The transfer of the lactyl group from lactyl-CoA to lysine residues on substrate proteins depends on a class of enzymes known as lactyltransferases. At the time of lactylation’s initial discovery, p300/CBP were considered the primary lactyltransferases. Zhang et al. (2019) demonstrated in their original report that p300 overexpression enhanced histone lactylation levels, whereas p300 knockdown significantly attenuated lactylation signal intensity [7]. Subsequent studies further confirmed the involvement of p300/CBP in the lactylation of HMGB1, with its inhibitor C646 effectively blocking this process [19].

However, the central role of p300 as a lactyltransferase is increasingly being challenged. Structural biology studies have revealed that p300’s preference for acyl-CoA substrates diminishes markedly with increasing acyl chain length; compared to acetyl-CoA, the transfer efficiency of p300 toward propionyl-CoA, butyryl-CoA, and crotonyl-CoA declines to approximately 33%, 2.2%, and 1.5%, respectively. Given that lactyl-CoA concentrations are two orders of magnitude lower than those of acetyl-CoA, the catalytic contribution of p300 to lactylation under physiological conditions may be rather limited [16]. This tension between theoretical predictions and experimental observations has catalyzed the search for novel lactyltransferases.

Based on sequence and structural homology, researchers turned their attention to the MYST family of acetyltransferases. Niu et al. (2024) demonstrated through molecular docking that HBO1 (also known as KAT7) exhibits superior binding affinity for lactyl-CoA compared to males absent on the first (MOF), tat interacting protein 60 kD (TIP60), and monocytic leukemia zinc finger protein (MOZ) [20]. In vitro enzymatic assays confirmed that HBO1 can directly catalyze histone H3K9 lactylation, with its catalytic activity completely abolished upon mutation of the key residue E508 to glutamine. Quantitative proteomics in HBO1-knockout HeLa cells identified 95 endogenous lactylation sites dependent on HBO1, the vast majority of which were located on nuclear proteins. Genome-wide CUT&Tag analysis further revealed that HBO1-mediated H3K9la is highly enriched at transcription start site (TSS) regions, directly regulating the transcription of tumor-associated genes such as AQP1, LAMC2, and F10. Notably, the lactyltransferase activity of HBO1 is positively modulated by its scaffold proteins JADE1 and BRPF2 [9,20]. In addition to HBO1, KAT8 (also known as MOF) has been confirmed to possess lactyltransferase activity, catalyzing lactylation at K408 of eEF1A2, enhancing its GTPase activity and promoting protein synthesis. ATAT1 and HDAC6 have also been shown to exhibit lactyltransferase functions under specific conditions [16,23].

#### 2.2.3. The Lactyl-CoA-Independent Pathway

**Figure 2:**
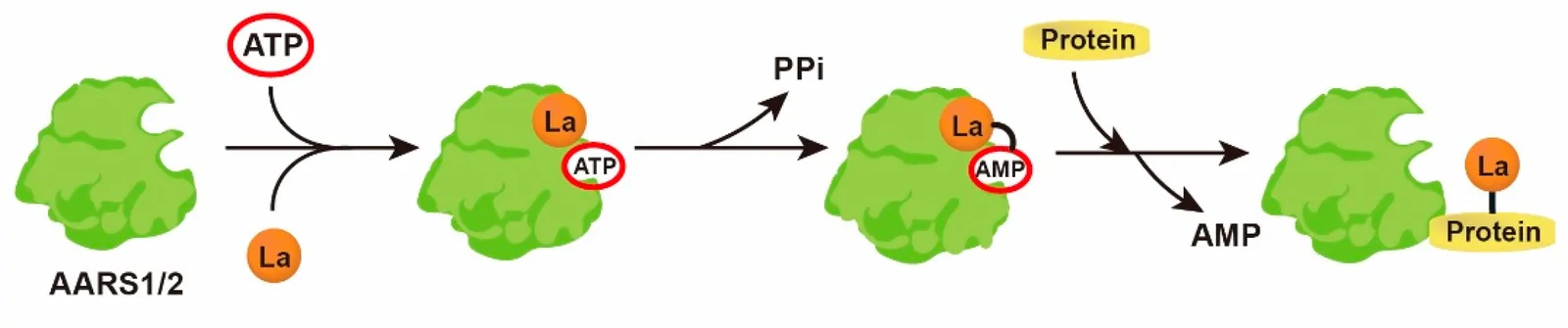
The process of AARS1/2-mediated lactylation in target proteins.

The fact that lactyl-CoA concentrations are substantially lower than those of acetyl-CoA has prompted the field to re-evaluate the donor sources for lactylation. In 2024, two independent studies jointly uncovered a lactylation pathway that entirely bypasses lactyl-CoA, identifying alanyl-tRNA synthetase AARS1 and its mitochondrial homolog AARS2 as novel lactate sensors and lactyltransferases [16,21,22]. Figure 2 illustrates the lactyl-CoA-independent lactylation pathway catalyzed by AARS1/2 (Figure 2). In the presence of ATP, AARS1/2 directly bind lactate to form a lactate-AMP intermediate, subsequently transferring the lactyl group to substrate lysines. This two-step reaction requires only lactate and ATP, independent of lactyl-CoA. Structural and mutational experiments have identified conserved lactate-binding residues (AARS1: M46, R77, N216, D239, G241; AARS2: M79, R110, N242, D265, G267), with this recognition mechanism being highly conserved from Escherichia coli to humans. The AARS1-R77Q mutation, frequently detected in gastric cancer, enhances its catalytic capacity [29].

AARS1/2-mediated lactylation covers a broad substrate spectrum. Genome-wide clustered regularly interspaced short palindromic repeats (CRISPR) screening and quantitative lactylomic analyses have demonstrated that the knockdown of either enzyme almost completely abolishes L-lactate-induced lactylation signals. AARS1 favors cytosolic and nuclear substrates, whereas AARS2 predominantly regulates the mitochondrial proteome, such as catalyzing lactylation at K131 (human)/K156 (mouse) of the N-terminus of cGAS, disrupting its phase separation capacity and DNA-binding activity to suppress the cGAS-STING pathway. Under hypoxic conditions, it catalyzes the lactylation of PDHA1 and CPT2, inhibiting oxidative phosphorylation [29,30]. In gastric cancer, AARS1 mediates the lactylation of YAP-K90 and TEAD1-K108, activating Hippo pathway target genes, with AARS1 itself serving as a target gene of YAP-TEAD to establish a positive feedback loop [22,29].

In summary, conventional acetyltransferases represented by p300/CBP, MYST family members including HBO1 and KAT8, and the novel lactate sensors/transferases exemplified by AARS1/2 collectively constitute a multi-layered, multi-mechanistic lactyltransferase network. The ACSS2-KAT2A and GTPSCS-p300 pathways rely on lactyl-CoA, whereas AARS1/2, localized to distinct subcellular compartments, directly utilize lactate and ATP for in situ catalysis. The relative contributions of individual enzymes may differ markedly across cell types, metabolic states, and stress conditions. Furthermore, as these enzymes simultaneously participate in multiple acylation modifications including acetylation, propionylation, and crotonylation, future studies should aim to delineate their division of labor and cooperative relationships through systematic comparisons and conditional knockout animal models.

### 2.3. The Delactylases

As a reversible post-translational modification, the dynamic equilibrium of lactylation depends not only on the catalytic addition by lactyltransferases but also on the selective removal of lactyl groups by delactylases. Given that both lactation and acetylation have similar chemical structures involving lysine ε-amino acylation modifications, researchers initially hypothesized that known lysine deacetylases (KDACs) might also possess delactylase activity. This hypothesis was subsequently validated through systematic experiments, which further revealed the multi-layered nature and substrate selectivity of the delactylase machinery.

#### 2.3.1. HDAC1-3 Are the Core Delactylases

Moreno-Yruela et al. (2022) systematically screened all 18 human lysine deacetylases and found that HDAC1-3 exhibit robust removal activity toward both L- and D-lactylated lysines. HDAC3 is the most potent among the three, showing only a 3.5-fold difference in catalytic efficiency between L- and D-lactylated substrates, whereas HDAC1/2 exhibit a preference for D-lactylation (>6-fold) [8]. Structural modeling revealed that the H134 residue in the active site of HDAC3 can form a hydrogen bond with the hydroxyl group of the D-lactyl moiety, a stereospecific interaction that may account for its stereoselectivity. On intact histones, HDAC1-3 substantially reduce global Kla levels as well as site-specific lactylation signals at H3K18la and H4K5la, as quantitative mass spectrometry detected 39 Kla peptides on the histone, of which 31 showed a reduction of over 90% in response to HDAC3 in at least one replicate. At the cellular level, broad-spectrum HDAC inhibitors and the HDAC1-3-specific inhibitor apicidin significantly elevated histone Kla levels, whereas class IIa HDAC inhibitors and NAM had no such effect, confirming that HDAC1-3 constitute the primary delactylase system in the nucleus. Overexpression of HDAC1 and HDAC3 significantly reduced H4K5la, whereas H3K18la remained relatively insensitive, suggesting that different HDAC isoforms exert selective regulatory effects on distinct histone sites [8,31]. The substrate spectrum of HDAC1-3 is not confined to histones but also encompasses non-histone proteins, including p53 K382, NF-κB p65, and α-tubulin [26,31].

#### 2.3.2. SIRT1-3 Is an Auxiliary Regulator of Delactylation

The roles of the SIRT family in delactylation are more complex and exhibit site dependence. In vitro screening demonstrated that SIRT1-3 possess histone Kla delactylase activity, albeit with catalytic efficiencies approximately 1000-fold lower than those of HDAC1-3 [8]. Fan et al. (2023) focused on H4K16la and found that SIRT3 exhibits a binding affinity (76.3 μM) significantly superior to that of SIRT1-2 [27]. Crystal structure analyses elucidated that F180, I230, I291, and V292 constitute a hydrophobic cage accommodating the hydrocarbon moiety of the lactyl group, while Q228, N229, and H248 stabilize the hydroxyl group through a water-mediated hydrogen bond network. SIRT3 exhibits approximately twofold higher affinity for D-lactylation than for L-lactylation. At the cellular level, SIRT3 knockout or treatment with the inhibitor 3-TYP significantly elevated H4K16la levels, whereas H4K12la was minimally affected [26,27].

Du et al. (2024), through the construction of SIRT1- and SIRT3-knockout HepG2 cell lines coupled with global lactylomic profiling (identifying 8075 Kla sites), demonstrated that SIRT1 regulates 1348 Kla sites (724 proteins), whereas SIRT3 regulates 1143 sites (680 proteins), with minimal overlap between their respective substrate pools, suggesting functional non-redundancy [24]. Gene Ontology enrichment revealed that SIRT1 substrates are enriched in RNA metabolism, whereas SIRT3 substrates are enriched in chromosome localization and DNA damage checkpoint pathways. PKM2 K207la, a specific substrate of SIRT1, is located within the ATP-binding pocket, impeding tetramer formation and reducing pyruvate kinase activity, thereby suppressing glycolysis and cell proliferation. ENO1 K228la was identified as a shared substrate of both enzymes, and SIRT2 has been reported to remove lactylation from METTL16, thereby regulating cuproptosis [23]. Collectively, the SIRT family exhibits functional specificity at particular substrate sites (such as PKM2 K207, H4K16, and METTL16 K229) that cannot be fully compensated for by HDAC1-3 [24,27,32].

Chen et al. (2025), employing a Protein A-TurboID proximity labeling strategy coupled with quantitative mass spectrometry, identified SIRT3 as a highly enriched H3K9la-binding protein, with a binding affinity of 14.0 μM. Mutation of key residues H248, D346, and V324 substantially weakened this interaction. In vitro experiments confirmed that SIRT3 removes H3K9la in an NAD^+^-dependent manner, with the H248Y catalytic mutant completely abolishing this activity. In esophageal squamous cell carcinoma (ESCC) cells, both SIRT3 knockout and treatment with the inhibitor 3-TYP significantly elevated H3K9la levels, accompanied by transcriptional activation of oncogenes including MYC, LAMC2, TGFA, and LAMB3, as well as enhanced proliferation and migration. ChIP-seq analysis demonstrated that SIRT3-regulated H3K9la is enriched at TSS regions, with its depletion leading to upregulation of 6065 genes primarily enriched in tumorigenesis-related pathways. In vivo, SIRT3-knockout KYSE30 cells exhibited significantly enhanced tumorigenic capacity in nude mice [26]. The Cancer Genome Atlas (TCGA) data analysis revealed that low SIRT3 expression in ESCC correlates with poor prognosis, and SIRT3 also suppresses hepatocellular carcinoma progression through delactylation of CCNE2 K348la [28].

In summary, delactylation exhibits a multi-layered network architecture. HDAC1-3 constitute the core delactylase system, maintaining global Kla homeostasis with broad substrate spectra and high catalytic efficiency. SIRT1-3 serve as an auxiliary regulatory layer, exerting irreplaceable functions at specific substrate sites. This architecture accounts for the differential kinetics of lactylation across cell types, and the NAD^+^ dependence of SIRT1-3 directly couples lactylation regulation to cellular energy status. The differential substrate preferences between HDACs and SIRTs suggest that they predominantly govern the transcriptional regulation and functional modulation of metabolic enzymes/signaling proteins, respectively. The coordinated action of delactylases and lactyltransferases constitutes a complete regulatory cycle, endowing lactylation with high spatiotemporal specificity and plasticity.

### 2.4. Substrate Diversity of Lactylation

The substrate repertoire of lactylation exhibits a bipartite structure of histones and non-histone proteins, which differ systematically in distribution breadth, stoichiometry, catalytic mechanisms, and modes of functional output [33].

In terms of distribution, the number of histone lactylation sites is far lower than that of non-histone sites. Zhang et al. (2019) identified 28 histone Kla sites in HeLa cells and BMDMs, among which H3K18la, H3K23la, H4K8la, and H4K12la have been the most extensively studied [7,34,35]. In contrast, a single lactylomic analysis of hepatocellular carcinoma tissues identified over 9275 Kla sites, of which 9256 are non-histone sites [36,37]. This disparity not only reflects detection sensitivity but also suggests that histone lactylation is constrained by nucleosome spatial restrictions and multi-site competition.

At the catalytic level, both share KAT families, including p300/CBP, HBO1, and KAT8, as well as the delactylases HDAC1-3 and SIRT1-3 [8,20]. However, AARS1/2 exhibit a significantly higher preference for non-histone substrates, such as p53 K120/K139 lactylation, attenuating its tumor-suppressive function [37], and AARS2 targeting PDHA1 and CPT2 [36]. This suggests that histone lactylation is more dependent on the classical KATs-lactyl-CoA pathway, whereas non-histone lactylation more frequently involves the AARS1/2-mediated independent pathway.

In terms of functional output, histone lactylation regulates gene transcription by altering chromatin accessibility and transcription factor recruitment. For instance, H3K18la directly regulates downstream target genes, including Ythdf1 and ARG1, and H4K8la drives fibroblast activation protein (FAP) transcription to mediate CD8^+^ T cell immune exclusion. Non-histone lactylation, by contrast, achieves rapid functional remodeling by altering enzymatic activity (PKM2 K62la), protein stability (DCBLD1 K172la), subcellular localization (ABCF1 K430la), and protein–protein interaction networks (MOESIN K72la) [35,36,37,38]. This division of labor between slow epigenetic regulation and rapid functional remodeling enables lactylation to respond to metabolic signals across different temporal scales.

In summary, histone lactylation is characterized by limited sites and epigenetic output, whereas non-histone lactylation is characterized by vast substrates and functional remodeling. The two are tightly coupled through shared enzymatic systems and substrate crosstalk, collectively constituting the complete pathway through which lactate signals are transduced from metabolic states to cellular functional outputs.

### 2.5. Dynamic Regulation and Metabolic Sensing of Lactylation

The dynamic equilibrium of lactylation is fundamentally a direct response to intracellular lactate concentrations, and fluctuations in this metabolite abundance are translated, through immediate adjustments of the enzymatic network, into reversible lysine chemical modifications.

Intracellular lactate levels constitute the primary determinant of lactylation extent. Under conditions of hypoxia, inflammation, or high glucose, upregulated glycolytic flux leads to significantly increased lactate production, thereby driving elevated lactylation levels [1]. Under hypoxia (1% O_2_), LDHA/LDHB-mediated lactate production serves as a prerequisite for HIF-1α protein accumulation, and the knockdown of LDHA and LDHB completely blocks hypoxia-induced increases in HIF-1α, whereas exogenous lactate supplementation restores this effect [39]. In sepsis-associated acute kidney injury, SIRT3 downregulation leads to PDHA1 hyperacetylation and inactivation, promoting excessive lactate production, which in turn drives Fis1 K20 lactylation, inducing mitochondrial hyperfragmentation and cellular injury [40]. Lactylation can convert transient metabolic signals into sustained functional outputs.

The competitive relationship between lactylation and other acylation modifications, such as acetylation, constitutes another critical dimension of lactylation dynamics. Both share the same lysine ε-amino group and partial enzymatic machinery (p300/CBP, HDAC1-3/SIRT1-3), subjecting the same lysine residue to competitive occupancy by multiple acylation modifications [41]. At the H4K12 site, the dynamic interconversion between lactylation and acetylation participates in the regulation of meiotic maturation in mouse oocytes [36]. PDHA1 K385 is subject to both acetylation and lactylation, and SIRT3 downregulation leads to PDHA1 hyperacetylation and inactivation, promoting lactate accumulation, which in turn drives Fis1 lactylation [40].

The pathophysiological significance of lactylation dynamics has been validated across multiple systems. In the cardiovascular system, exercise-induced lactate increases inhibit atherosclerosis through MECP2 K271 lactylation, whereas pathological lactate accumulation drives myocardial fibrosis through Snail1 lactylation. In the tumor microenvironment, lactate drives CPT1A transcription through H3K18la while simultaneously inhibiting its ubiquitin-proteasome degradation through CPT1A protein K180/K285 lactylation. In adipose tissue, insulin-stimulated glucose uptake promotes lactate release from adipocytes, which activates GPR81 in an autocrine manner, inhibiting adenylate cyclase and reducing cAMP production to mediate the antilipolytic effect of insulin [36,40,42].

The dynamic regulation of lactylation does not simply follow a linear logic that more lactate needs more modification. pH changes, cofactor availability (such as the NAD^+^/NADH ratio), and the expression and activity states of enzymes all constitute regulatory nodes [1]. SIRT3 activity is directly influenced by cellular energy status, while AARS1/2 depend on ATP availability [36,39]. The presence of these multi-layered regulatory nodes enables the same lactate level to produce differential modification profiles and functional consequences across cell types or pathological states.

In summary, the dynamic regulation of lactylation operates with intracellular lactate concentration as the core variable and tightly couples multiple metabolic dimensions, including glycolytic flux, mitochondrial function, NAD^+^ metabolism, and acyl-CoA pools, positioning lactylation as both a sensitive indicator and a functional executor of cellular metabolic states. Its dysregulation in tumors, inflammation, ischemia, and metabolic diseases may serve as a driving force for pathological progression.

## 3. Functions of Lactylation in Core Cancer Hallmarks

To elucidate the functions of lactylation in the malignant progression of tumors, this section adopts the perspective of core cancer hallmarks and integrates current multi-layered evidence on how lactylation participates in key pathological processes, including tumor cell proliferation and survival, metabolic reprogramming, cell death dysregulation, and invasion and metastasis. Unlike the linear influence of a single metabolite or a single modification event, lactylation constructs a complex regulatory network through direct modification of metabolic enzyme activity, remodeling of damage-associated molecular pattern secretion, driving histone-mediated epitranscriptional regulatory axes, and regulating mitochondrial dynamics and the immune microenvironment. The following sections will systematically review the functional logic and molecular basis of lactylation in core cancer hallmarks across four dimensions: proliferation and survival, metabolic remodeling, cell death regulation, and metastasis and invasion (Table 2).

### 3.1. Tumor Cell Proliferation and Survival

Lactylation supports tumor cell proliferation and survival through multiple levels of regulation, including direct modulation of metabolic enzyme activity, remodeling of the secretory fate of damage-associated molecular patterns, and driving epigenetic transcriptional regulatory axes, collectively constituting a regulatory network through which lactylation promotes malignant proliferation.

At the level of direct metabolic enzyme modification, lactylation at K62 of PKM2 represents a key node for understanding how lactylation feeds back to regulate glycolytic flux. In lipopolysaccharide-stimulated inflammatory macrophages, Wang et al. (2022) found that lactate catalyzes PKM2 K62 lactylation through p300/CBP. This modification enhances PKM2 enzymatic activity while inhibiting its transition from tetramer to dimer and nuclear translocation, thereby attenuating the capacity of PKM2 to regulate glycolytic genes as a transcriptional coactivator [43]. Functionally, PKM2 K62la in inflammatory macrophages serves as a negative feedback mechanism to limit excessive glycolysis and prevent metabolic exhaustion. Although this finding originates from macrophage models, high PKM2 expression in hepatocellular carcinoma has been confirmed to be significantly associated with poor patient survival, and prognostic analyses based on lactylation-related gene signatures have identified PKM2 as a key component of the model [64]. This clinical association suggests that the function of PKM2 lactylation in tumors may be context-dependent. In inflammatory cells, it restricts glycolysis to maintain metabolic homeostasis, whereas in tumor cells, it may cooperate with other lactylating enzymes to sustain the proliferative advantage driven by the Warburg effect. However, its specific effects in tumors require further validation.

At the level of damage-associated molecular pattern (DAMP) molecule modification, lactylation of HMGB1 reveals how metabolic signals are directly transduced into proliferation and pro-inflammatory outputs. Yang et al. (2022) demonstrated in a sepsis model that macrophages take up extracellular lactate via monocarboxylate transporters, which directly mediates lysine lactylation of HMGB1 catalyzed by p300/CBP. Concurrently, lactate triggers inhibition of the Hippo/YAP pathway through the GPR81 receptor. Lactate induces LATS1-mediated YAP phosphorylation and degradation, thereby relieving YAP-mediated maintenance of SIRT1 transcription, resulting in decreased SIRT1 and promoting HMGB1 acetylation. On the other hand, lactate recruits p300/CBP acetyltransferases to the nucleus in a β-arrestin2-dependent manner. The synergistic action of lactylation and acetylation drives HMGB1 translocation from the nucleus to cytosolic lysosomes, ultimately releasing it in the form of exosomes. Released HMGB1-containing exosomes disrupt endothelial cell VE-cadherin and Claudin5 integrity and upregulate ICAM1 expression, leading to increased vascular permeability [19].

At the level of epigenetic transcriptional regulatory axes, histone lactylation indirectly regulates immune cell survival and function through remote control of RNA-modifying enzyme transcription. Qian et al. (2025) found that H3K18la levels are significantly elevated in gastric cancer tissues and negatively correlated with CD8^+^ T cell infiltration. Mechanistically, H3K18la directly binds to the METTL3 promoter region and drives its transcriptional upregulation. METTL3 stabilizes CCT2 mRNA and enhances its protein expression through m6A modification. CCT2 suppresses CD8^+^ T cell proliferation and the expression of key cytotoxic factors, including IFN-γ, TNF-α, and granzyme B, by inhibiting Ca^2+^ influx. The LDH inhibitor oxamate reverses this immunosuppressive effect and enhances CD8^+^ T cell antitumor activity by reducing lactate production and H3K18la levels. In tumor-bearing mice, METTL3 knockdown combined with oxamate treatment synergistically inhibited tumor growth, accompanied by increased intratumoral CD8^+^ T cell infiltration [44].

Through these three lines of evidence, the promotion of tumor proliferation and survival by lactylation is not a linear output of a single mechanism but is achieved through functional coupling across multiple levels. PKM2 K62la constitutes a fast reaction layer of metabolite feedback regulation, while HMGB1 lactylation and acetylation constitute a signal output layer of DAMP release. H3K18la, METTL3 and CCT2 together constitute a durable adaptation layer of epigenetic reprogramming. Lactate serves as the upstream metabolic signal, yet these mechanisms exhibit fundamental differences in intracellular self-restriction, intercellular signal output, and cross-cell immunosuppression. Together, they constitute a mechanistic network that supports the sustained proliferation of tumor cells under metabolic stress and immune pressure.

### 3.2. Metabolic Reprogramming

Metabolic reprogramming is a core adaptive change that enables tumor cells to meet the demands of proliferation. Lactylation plays a dual role in this process. One major role is that lactate accumulation, driven by increased glycolytic flux, serves as the direct substrate source for lactylation. Another significant role is that lactylation reinforces glycolysis, suppresses oxidative phosphorylation, and remodels lipid and glutamine metabolism by modifying histones and non-histone proteins. This establishes a positive feedback regulatory network in which metabolism and epigenetics mutually amplify each other. This section systematically elaborates on how lactylation drives tumor metabolic reprogramming from five dimensions: glycolysis positive feedback, oxidative phosphorylation suppression, lipid metabolism remodeling, glutamine metabolism regulation, and mitochondrial dynamics.

#### 3.2.1. Lactylation-Driven Glycolysis Positive Feedback Loop

Elevated glycolytic flux leads to lactate accumulation, and lactate in turn further upregulates the expression or activity of glycolysis-related enzymes through lactylation, forming a self-reinforcing cycle in which glycolysis is enhanced, lactate accumulates, lactylation is exacerbated, and glycolytic genes are further activated. The molecular basis of this positive feedback lies in the direct transcriptional regulation of glycolytic enzyme genes by histone lactylation. In non-small cell lung cancer, exogenous lactate downregulates the expression of key glycolytic enzymes, including HK1, G6PD, and pyruvate kinase M (PKM), through histone lactylation modifications such as H4K8la, while simultaneously upregulating tricarboxylic acid cycle enzymes, including succinate dehydrogenase (SDH) and isocitrate dehydrogenase (IDH), thereby remodeling metabolic flux distribution [45]. In papillary thyroid carcinoma, lactate enhances CPT1A transcription through H3K18la enrichment at its promoter region, while lactylation at K180 and K285 of the CPT1A protein itself inhibits its ubiquitin-proteasome degradation. These two layers—transcriptional activation and protein stabilization—synergistically amplify fatty acid oxidation flux and drive tumor migration [42].

The lactate-driven glycolysis positive feedback loop operates not only within tumor cells but can also be extended through metabolic coupling among cells in the microenvironment. In pancreatic cancer, nerve involvement with associated fibroblasts enhances GAPDH K66 acetylation through the TGFβ-mTOR-AKT-P300 axis, increasing its enzymatic activity and driving high glycolytic flux with substantial lactate secretion into the microenvironment. Tumor cells take up this exogenous lactate via MCT1, which triggers H3K18la in the nucleus and subsequently transcriptionally activates perineural invasion genes, including L1CAM and SLIT1 [63]. Notably, this mechanism depends on GAPDH K66 acetylation in pCAFs enhancing its enzymatic activity, illustrating the cascade collaboration between acetylation and lactylation in metabolic regulation, where acetylation events within fibroblasts drive lactate production, which in turn drives lactylation events within tumor cells.

In the context of drug resistance, the reinforcement of the glycolysis positive feedback is particularly prominent. In hepatocellular carcinoma cells resistant to oxaliplatin and 5-fluorouracil, glycolytic activity is significantly enhanced, accompanied by markedly elevated lactate levels and specific enrichment of H3K14la. Chromatin immunoprecipitation sequencing revealed that H3K14la directly binds to the NEDD4 promoter and drives its transcription. NEDD4, as an E3 ubiquitin ligase, promotes ubiquitination and degradation of phosphatase and tensin homolog (PTEN), thereby relieving PTEN-mediated inhibition of the PI3K-AKT-mTOR pathway and further driving glycolysis, forming a closed pathway [46]. In diffuse large B-cell lymphoma, P300 enriches H3K18la at the HK2 promoter region and activates its transcription, enhancing glycolytic flux and lactate production. Lactate in turn further drives H3K18la, promoting doxorubicin resistance. This finding directly links drug resistance to positive feedback regulation, indicating that lactylation is not merely a consequence of metabolism but an active driver of resistance maintenance.

In lung cancer, TOP2A has been identified as an upstream regulator of LDHA. TOP2A knockdown reduces the extracellular acidification rate, glucose uptake, and lactate production, while also decreasing global lysine lactylation and H3K18la levels. TOP2A overexpression partially rescues the inhibitory effects of glycolysis inhibitors on these parameters. The positive regulation of TOP2A on LDHA depends on the activation of the MEK-ERK pathway, while lactate produced by LDHA maintains TOP2A expression through histone lactylation, forming a closed pathway [47]. Integrative analysis of multiple databases identified NCAPD3 as a lactylation-related prognostic gene. Its expression promotes glycolysis in lung cancer through the same pathway, while histone lactylation in turn sustains NCAPD3 expression [48]. These findings collectively demonstrate a pathway in which initiating signals (oncogenes, drug resistance pressure, or microenvironmental factors) drive LDHA and glycolytic enzyme upregulation, leading to lactate accumulation, which in turn triggers histone lactylation, promotes further transcriptional activation of glycolytic genes, and generates more lactate.

#### 3.2.2. Suppression of Oxidative Phosphorylation

Alongside the enhancement of glycolysis, lactylation also participates in suppressing oxidative phosphorylation, driving tumor cells toward a glycolysis dependency. This effect is primarily achieved through the modification of mitochondrial metabolic enzymes. Under hypoxic conditions, AARS2 accumulates in the mitochondria and catalyzes lactylation of PDHA1 (K336) and CPT2 (K457 and K458), inhibiting the activity of the pyruvate dehydrogenase complex and carnitine palmitoyltransferase 2, thereby limiting mitochondrial oxidative utilization of pyruvate and fatty acids [30]. This mechanism reveals a complete signaling chain from hypoxia to AARS2 upregulation to mitochondrial protein lactylation and ultimately to oxidative phosphorylation suppression, directly coupling microenvironmental oxygen sensing to mitochondrial metabolic regulation. In sepsis-associated acute kidney injury, SIRT3 downregulation leads to PDHA1 K385 hyperacetylation and inactivation, promoting excessive lactate production. Lactate in turn drives Fis1 K20 lactylation, promoting mitochondrial hyperfragmentation and energy depletion [40]. Although this study primarily focused on renal tubular epithelial cells rather than tumor cells, the chain of PDHA1 inactivation, lactate elevation, Fis1 lactylation, and mitochondrial dysfunction it revealed suggests that the effect of lactylation in suppressing oxidative phosphorylation may be conserved across cell types.

Lactylation of HIF-1α participates in oxidative phosphorylation suppression and metabolic reprogramming at the transcriptional regulation level. Li et al. (2025) found that lactate induces HIF-1α lactylation at different sites across species (mouse K644, human and pig K12). This modification does not rely on the classical proline hydroxylation pathway but directly blocks Von Hippel–Lindau tumor suppressor (VHL) recognition of HIF-1α through steric hindrance, thereby stabilizing HIF-1α protein and enhancing its transcriptional activity, including upregulation of glycolytic genes Vegfa and Glut1 and suppression of oxidative phosphorylation-related gene expression. AlphaFold3 structural modeling revealed that K12 and K644 lactylation induce conformational changes in HIF-1α, shortening the distance between key hydroxylation sites and modification sites, revealing the molecular basis by which distal lactylation regulates protein–protein interactions through allosteric effects [39]. The suppression of oxidative phosphorylation by lactylation is not a simple inhibitory effect but rather acts in synergy with glycolysis enhancement. Lactylation-mediated suppression of oxidative phosphorylation further reinforces dependence on glycolytic energy supply, while enhanced glycolysis provides additional lactate substrate, forming a bidirectional reinforcement system for metabolic reprogramming.

#### 3.2.3. Remodeling of Lipid Metabolism

Aberrant activation of lipid metabolism constitutes an important component of tumor metabolic reprogramming, and lactylation plays a critical role in this process. In KRAS-mutant colorectal cancer, the mutant Kirsten rat sarcoma viral oncogene homolog (KRAS) elevates intracellular lactate levels through enhanced glycolysis, thereby driving H3K9la enrichment at the promoter region of the cholesterol transporter GRAMD1A. H3K9la increases chromatin accessibility at this region and promotes GRAMD1A transcription. GRAMD1A in turn promotes SREBP2 nuclear translocation and activation, upregulating the expression of cholesterol synthesis rate-limiting enzymes, including 3-hydroxy-3-methylglutaryl-CoA reductase (HMGCR), and driving cholesterol metabolic reprogramming as well as tumor growth and metastasis. In patient-derived xenograft (PDX) models, both LDH inhibitors and GRAMD1A inhibitors significantly suppressed tumor growth in KRAS-mutant colorectal cancer, with combination therapy showing even greater efficacy, suggesting the therapeutic potential of targeting the lactylation–lipid metabolism axis [49]. In pancreatic cancer, the deubiquitinase PSMD14 directly binds to and deubiquitinates LDHA, extending its half-life, stabilizing LDHA protein levels, and promoting lactate production. Elevated lactate drives H3K18la enrichment at the ATP citrate lyase (ACLY) gene enhancer region, enhancing ACLY transcription and protein expression, thereby promoting fatty acid synthesis and lipid accumulation. Combination therapy with the PSMD14 inhibitor thiolignan and the LDHA inhibitor oxamate synergistically suppressed tumor growth in PDX models, supporting the therapeutic strategy targeting the PSMD14-LDHA-lactate-H3K18la-ACLY pathway [50]. Notably, PSMD14 regulation of LDHA occurs at the protein stability level rather than the transcriptional level, suggesting that in the tandem between metabolism and appearance, non-histone lactylation (as a downstream effect of lactate in this case) and deubiquitination (stabilization of LDHA) represent parallel rather than mutually exclusive regulatory layers.

In a sorafenib-resistant hepatocellular carcinoma model, H3K18la was identified as a key epigenetic modification driving lipid metabolic reprogramming. Elevated lactate in resistant cells drives H3K18la enrichment at the HOXB13 promoter region and activates its transcription, and then HOXB13 activates HIF-1 signaling, thereby driving lipid synthesis and lipid droplet accumulation [51]. Both HOXB13 knockdown and HIF-1 pathway inhibition reversed lipid accumulation. In papillary thyroid carcinoma, H3K18la drives CPT1A transcription, while K180 and K285 lactylation inhibits CPT1A degradation, synergistically amplifying fatty acid oxidation flux and providing energy support for tumor metastasis [42]. CPT1A lactylation site mutations (K180R and K285R) significantly attenuated tumor migration and growth both in vitro and in vivo, validating the functional importance of non-histone lactylation in lipid metabolism regulation.

These findings collectively indicate that lactylation regulates lipid metabolism at two levels. Histone lactylation activates the transcription of lipid synthesis and transport-related genes by remodeling the chromatin environment, while non-histone lactylation modulates the activity and stability of metabolic enzymes such as CPT1A through direct modification. The two levels synergistically constitute a complete regulatory axis.

#### 3.2.4. Regulation of Glutamine Metabolism

Glutamine is the most important carbon and nitrogen source for tumor cells after glucose. Its catabolism provides key precursors for tricarboxylic acid cycle anaplerosis, nucleotide synthesis, and lipid production. The regulation of glutamine metabolism by lactylation has begun to emerge, primarily involving two aspects. Lactylation upregulates the expression of glutamine transporters and catabolic enzymes through epigenetic mechanisms, while glutamine metabolism itself reciprocally regulates lactylation by influencing lactate levels.

The transcription of the glutamine transporter SLC6A14 is directly regulated by H3K18la. In bladder cancer, hypoxia-induced PYCR1 enhances lactate production through increased glycolysis, driving H3K18la enrichment at the SLC6A14 promoter region and upregulating its expression. SLC6A14, as a broad-spectrum amino acid transporter with a high affinity for glutamine, significantly enhances glutamine uptake and downstream catabolism upon upregulation, including conversion of glutamine to glutamate and accumulation of α-ketoglutarate, thereby promoting the proliferation, migration, and invasion of bladder cancer cells [52]. In tumor-bearing mice, PYCR1 knockdown reduced H3K18la levels and SLC6A14 expression, suppressing tumor growth and lung metastasis. SLC6A14 overexpression partially rescued this effect, establishing the complete signaling axis of PYCR1-glycolysis-H3K18la-SLC6A14-glutaminolysis.

Direct regulation of glutamine catabolic enzymes by lactylation is also worthy of attention. In pancreatic cancer, PSMD14 stabilizes LDHA through deubiquitination, promoting lactate accumulation and H3K18la. H3K18la not only activates ACLY but also upregulates the expression of glutamine metabolic enzymes, including glutaminase [50].

#### 3.2.5. Mitochondrial Dynamics

Mitochondria serve as the central hub for cellular energy metabolism and signal transduction. The dynamic balance of their morphology and function profoundly influences the metabolic adaptation of tumor cells. Lactylation participates in the regulation of mitochondrial network dynamic remodeling through modification of mitochondrial fission regulatory proteins.

**Figure 3:**
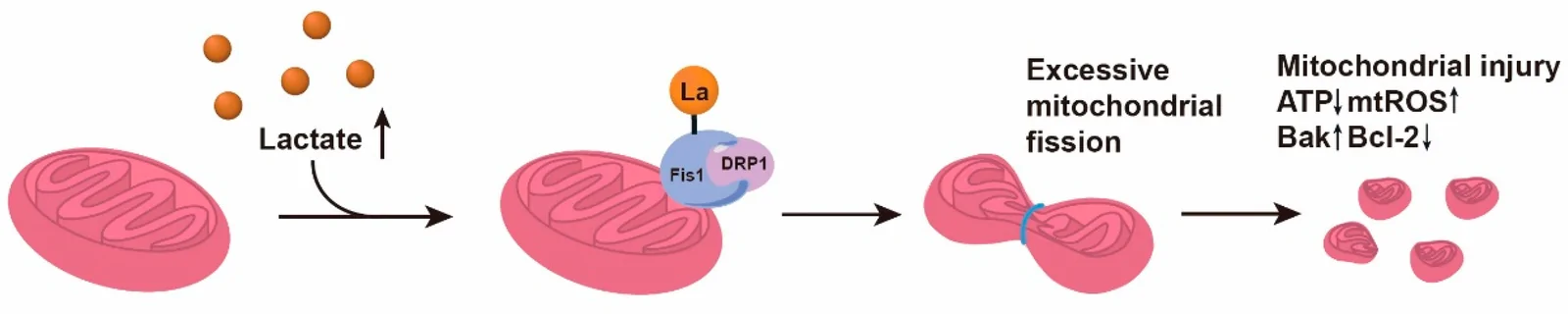
Lactate drives K20 lactylation of Fis1, enhancing the interaction between Fis1 and DRP1 and promoting excessive mitochondrial fission.

The activity of DRP1, the primary executor of mitochondrial fission, is indirectly influenced by upstream lactylation signals. In sepsis-associated acute kidney injury, lactate drives K20 lactylation of Fis1, a mitochondrial outer membrane fission adaptor protein, enhancing the interaction between Fis1 and DRP1 and promoting excessive mitochondrial fission. Lactylated Fis1-K20la exacerbates renal tubular epithelial cell injury by increasing mitochondrial fragmentation, depleting ATP, promoting mtROS production, and inducing mitochondrial apoptosis [40]. Figure 3 summarizes this regulatory axis (Figure 3). The Fis1 K20R mutation, which mimics delactylation, significantly weakens Fis1-DRP1 binding, inhibits mitochondrial fission, and rescues cell viability, demonstrating that Fis1 K20la is a critical regulatory node for mitochondrial fission. Although this study was conducted in the context of sepsis-associated kidney injury, Fis1, as a conserved mitochondrial outer membrane adaptor protein, may similarly influence mitochondrial dynamics and metabolic adaptation through its lactylation in tumor cells.

The activity of the mitochondrial fusion protein OPA1 is regulated by lactylation-related metabolic signals. SIRT3 activates OPA1 through deacetylation, promoting mitochondrial fusion [65]. Given that SIRT3 also possesses delactylase activity [27], a competitive relationship may exist between the acetylation and lactylation of OPA1, providing a new perspective for understanding the switch between mitochondrial fusion and fission under metabolic stress. Since lactylation and acetylation share enzymatic systems, including p300, lactylation may influence OPA1 activity through analogous mechanisms.

The balance between mitochondrial fission and fusion is also indirectly regulated by metabolite supply. Glutamine supplementation suppresses glycolysis and lactate production, reduces AMPKα lactylation levels, enhances its phosphorylation and activity, and thereby inhibits mitochondrial fission and promotes fusion through the AMPK-MFF pathway [66]. In an intervertebral disc degeneration model, glutamine-induced AMPKα delactylation promoted mitochondrial network fusion. This contrasts with the fission preference observed in tumor cells, suggesting that the regulation of mitochondrial dynamics by lactylation is context-dependent. In the tumor microenvironment, lactate-driven Fis1 lactylation promotes fission to accommodate rapid proliferation, whereas under nutrient-replete conditions with lower lactylation levels, fusion is favored to maintain energy efficiency. In tumors, mitochondrial fission is typically associated with invasiveness and chemoresistance [67], suggesting that targeting the lactylation–Fis1-DRP1 axis may represent a novel strategy to constrain tumor metabolic adaptation.

Therefore, the regulation of mitochondrial dynamics by lactylation primarily involves two levels. One is the direct promotion of mitochondrial fission through lactate-driven lactylation of fission adaptor proteins such as Fis1. The other is the indirect regulation of the balance between fusion and fission through metabolic signals. Research in this area remains in its early stages. Whether core dynamics proteins, including DRP1, MFN1/2, and OPA1, undergo direct lactylation modification, and how lactylation cooperates with other PTMs to regulate mitochondrial dynamics, will be important directions for future investigation.

### 3.3. Regulation of Cell Death by Lactylation

Dysregulation of cell death programs is a core element in tumorigenesis and therapeutic resistance. The discovery of lactylation modification provides a direct epigenetic and protein chemical basis for understanding how lactate regulates cell death. In recent years, the regulatory roles of lactylation in four major programmed cell death pathways—autophagy, pyroptosis, ferroptosis, and apoptosis—have been progressively elucidated, forming a regulatory axis from metabolic flux to lactate accumulation to lysine lactylation to death program reprogramming.

In the field of autophagy regulation, lactylation demonstrates multi-layered modulation of autophagic flux. In colorectal cancer, tumor-derived lactate enhances H3K18la enrichment at the promoter region of Rubicon-like autophagy enhancer (RUBCNL), transcriptionally activating the expression of this autophagy enhancer factor. RUBCNL interacts with BECN1 to promote the recruitment and functional activation of class III phosphatidylinositol 3-kinase complexes, accelerating autophagosome maturation into autolysosomes. This provides a survival advantage for tumor cells under hypoxic conditions and mediates resistance to bevacizumab therapy [53]. This finding links extracellular lactate signals, histone lactylation, and autophagic flux regulation into a complete molecular pathway. Meanwhile, in a sepsis model, lactate enters macrophages via monocarboxylate transporters and induces lactylation of the HMGB1 protein in a p300/CBP-dependent manner. Through GPR81 receptor-mediated signaling, lactate also promotes HMGB1 acetylation. The synergistic action of lactylation and acetylation drives HMGB1 translocation from the nucleus to the cytoplasm and subsequent extracellular release via exosomes, thereby disrupting vascular endothelial barrier function [19]. This study reveals a unique role of non-histone lactylation in the extracellular release of autophagy-related proteins. In castration-resistant prostate cancer, H3K18la and H4K12la enrichment at the CNN1 promoter region promotes CNN1 transcriptional upregulation. CNN1 overexpression confers resistance to docetaxel by modulating autophagy levels and G0/G1 cell cycle arrest. Inhibition of lactate production reduces CNN1 expression and restores drug sensitivity [54]. These findings indicate that lactylation regulates autophagy both at the histone level, by remodeling the transcriptional network of autophagy-related genes, and through direct modification of autophagy-related proteins to alter their subcellular localization and secretion behavior.

Pyroptosis, a pro-inflammatory programmed cell death, is also subject to lactylation regulation. In a bilirubin encephalopathy model, glycolysis enhancement in astrocytes under free bilirubin stimulation leads to lactate accumulation and elevated H3K18la levels. Genome-wide CUT&Tag combined with RNA-seq analysis identified nucleotide-binding oligomerization domain-containing protein 2 (NOD2) as a direct transcriptional target of H3K18la. H3K18la enrichment at the NOD2 promoter region promotes NOD2 expression, which in turn activates downstream MAPK and NF-κB signaling pathways, driving NLRP1-Caspase-1-GSDMD-IL-1β-dependent astrocyte pyroptosis and exacerbating neuroinflammation and brain injury [55]. In bladder cancer, mannose directly binds to PKM2 and inhibits its enzymatic activity, reducing lactate production and PKM2 K433 lactylation. Mannose competitively occupies the PKM2 active pocket to suppress its pyruvate kinase activity. K433 was identified as a key lactylation site by mass spectrometry, and the K433R mutation simultaneously abolishes lactylation and reduces PKM2 enzymatic activity. At the level of modification competition, mannose decreases K433 lactylation while increasing acetylation, accompanied by enhanced Y105 phosphorylation of PKM2, driving its translocation from the cytosol to the nucleus. Nuclear PKM2 enhances the transcription of NLRP1, Caspase-1, GSDMD, and IL-1β through direct binding to NF-κB (p50/p65), inducing pyroptosis and activating antitumor immune responses [56]. Notably, this study also revealed a competitive relationship between lactylation and acetylation at the same lysine residue (PKM2 K433). Mannose treatment reduces K433 lactylation while increasing acetylation, and this modification switch constitutes a molecular switch for PKM2 functional conversion from a metabolic enzyme to a transcriptional regulator. These findings collectively suggest that pyroptosis-related gene expression is subject to direct transcriptional regulation by histone lactylation, while the lactylation status of non-histone proteins can indirectly activate pyroptosis pathways by altering the subcellular localization of key signaling proteins.

In ferroptosis, lactylation exhibits an opposite regulatory direction. In non-alcoholic steatohepatitis, tRF-31R9J, a tRNA fragment derived from valine tRNA, directly binds to histone deacetylase HDAC1 and recruits it to the promoter regions of pro-ferroptotic genes ATF3, ATF4, and CHAC1, reducing H3K18la modification levels at these loci and thereby suppressing their transcription. This mechanism effectively reduces hepatocyte sensitivity to ferroptosis and ameliorates the pathological progression of steatohepatitis [57]. The innovation of this study lies in revealing a non-coding RNA regulatory axis from tRNA fragments to HDAC1 to histone delactylation to ferroptosis gene silencing, linking delactylation mechanisms to ferroptosis. Although the framework suggests that GSDMD lactylation remains unclear, existing evidence indicates that lactate can upregulate the expression of core negative regulators of ferroptosis, including GPX4 and SLC7A11, through histone lactylation, suggesting that lactylation may exert inhibitory functions in ferroptosis. The specific mechanisms require further exploration.

In the field of apoptosis, lactylation involvement primarily concerns p53 and the mitochondrial apoptosis pathway. p53, as the guardian of the genome, undergoes lactylation at K120 and K139, which has been confirmed to inhibit p53’s DNA binding capacity and transcriptional activity, thereby reducing the expression of pro-apoptotic genes, including Bax and Puma, and conferring apoptosis resistance to tumor cells. In glioblastoma, the interaction between SUCLG2 and lamin A, along with lamin A (LMNA) K470 acetylation, leads to decreased expression of the mitochondrial fusion protein MFN1, inducing mitochondrial fragmentation and reduced oxidative phosphorylation levels, which in turn triggers mitochondrial pathway apoptosis. In this process, SUCLG2 simultaneously interacts with dihydrolipoamide S-acetyltransferase (DLAT), promoting pyruvate conversion to acetyl-CoA, reducing lactate levels, and diminishing H4K16la binding to the promoters of genes, including BEST1, GRAMD4, and MBD6, further inhibiting glioblastoma proliferation [59]. This finding reveals a complex regulatory mode in which the same metabolic enzyme (SUCLG2) operates through two parallel molecular pathways, the LMNA-dependent mitochondrial apoptosis pathway and the DLAT-dependent lactylation epigenetic regulatory pathway, synergistically promoting tumor cell apoptosis.

### 3.4. Lactylation and Tumor Invasion and Metastasis

Evidence at the clinical translational level reinforces the association between lactylation and tumor metastasis. In pancreatic cancer patients, lactate levels, global lactylation, and H3K18la levels in PNI-positive tissues are significantly higher than in PNI-negative tissues, and high H3K18la expression is closely associated with shortened overall survival and disease-free survival [63]. In colorectal cancer, H3K9la levels are significantly elevated in KRAS-mutant tumors, and its expression positively correlates with clinical stage and distant metastasis [49]. In papillary thyroid carcinoma, lactate enhances fatty acid oxidation through dual mechanisms, H3K18la-dependent transcriptional activation and CPT1A protein K180/K285 lactylation-dependent stabilization, providing metabolic fuel for metastasis [42]. These clinical and animal model data collectively indicate that lactylation modification levels may serve as potential biomarkers for tumor invasion and metastatic potential.

Invasion and metastasis are the core processes underlying the lethal clinical manifestations of malignant tumors. Epithelial-mesenchymal transition (EMT), as a critical biological process through which tumor cells acquire migratory and invasive capacities, has long been a focus of mechanistic investigation. Lactate, as a metabolic product of elevated glycolytic flux, accumulates substantially in the tumor microenvironment. The discovery of lactylation modification has provided a novel molecular perspective for understanding how metabolic reprogramming drives EMT and metastasis. Current evidence indicates that lactylation can remodel EMT-related transcriptional networks and signaling pathway activities through both histone epigenetic regulation and direct non-histone protein modification, thereby promoting tumor invasion and metastasis.

At the level of histone lactylation, H3K18la has been independently identified by multiple studies as an upstream regulatory event for the transcriptional activation of EMT-related genes. In gastric cancer, H3K18la directly binds to the promoter region of hyaluronan synthase 2 (HAS2), activating its transcription. Upregulated HAS2 further promotes c-MYC nuclear translocation, which in turn binds to the CD274 (PD-L1) promoter and enhances its expression, thereby achieving immune evasion while driving EMT [60]. This study reveals a dual effect of lactylation through a single epigenetic modification event, simultaneously activating the invasive phenotype and the immunosuppressive microenvironment. In colorectal cancer, KRAS mutations elevate intracellular lactate levels through enhanced glycolytic flux, driving H3K9la enrichment at the GRAMD1A promoter region. GRAMD1A, as a cholesterol transporter, accelerates tumor growth and liver metastasis by promoting cholesterol metabolism, and this effect is particularly pronounced in KRAS-mutant tumors [49]. These findings link oncogene mutations, metabolic reprogramming, and epigenetic regulation into a complete pro-metastatic signaling chain.

The role of non-histone lactylation in EMT regulation is equally significant. In pancreatic cancer, the Rho GTPase family member Rho family GTPase F (RHOF) promotes PKM2 transcription through upregulation of c-Myc, enhancing glycolytic flux and lactate production. Lactate subsequently induces lactylation of the Snail1 protein, promoting its nuclear translocation and suppressing E-cadherin transcription, thereby driving EMT progression [61]. Notably, Snail1, as a core transcriptional repressor of EMT, undergoes lactylation that does not alter total protein levels but rather amplifies its transcriptional repressive function through enhanced nuclear localization signaling. This regulatory mode differs from the classical ubiquitin-proteasome degradation pathway. In gallbladder cancer, K183 lactylation of the YY1 transcription factor enhances its binding capacity to the FBXO33 promoter region, upregulating FBXO33 expression. FBXO33, as an E3 ubiquitin ligase, promotes p53 degradation by mediating polyubiquitination at K291/K292, thereby relieving p53-mediated inhibition of EMT [62]. This study extends the impact of lactylation from transcription factor activity to the regulation of tumor suppressor protein stability.

At the tumor microenvironment level, the regulation of EMT by lactylation is not confined to within tumor cells. In pancreatic cancer, cancer-associated fibroblasts (CAFs) enhance P300 phosphorylation and enzymatic activity through activation of the TGFβ/mTOR/AKT signaling pathway, thereby promoting GAPDH K66 acetylation, increasing its enzymatic activity and glycolytic flux. The substantial lactate secreted by pCAFs is taken up by tumor cells via MCT1, driving H3K18la enrichment at the L1CAM and SLIT1 promoter regions and activating the transcription of these two perineural invasion-associated genes, thereby promoting peripancreatic nerve invasion [63]. This discovery integrates cancer-associated fibroblast (CAF) metabolic reprogramming, lactate transport, and tumor cell epigenetic remodeling into a transcellular regulatory network, expanding the mechanistic dimension of lactylation in promoting invasion and metastasis.

## 4. Lactylation and the Tumor Immune Microenvironment (TME)

### 4.1. Lactylation Regulates Immune Checkpoints

Aberrant expression of immune checkpoint molecules is a core mechanism of tumor immune evasion. Multiple independent studies have firmly established that lactylation plays an important role in the expression regulation of immune checkpoints, particularly PD-L1, through both transcriptional and post-translational mechanisms.

At the transcriptional activation level, histone H3K18la has been independently confirmed by multiple studies as an upstream regulatory event for PD-L1 gene expression. In gastric cancer, lactate drives H3K18la enrichment at the HAS2 promoter region. Upregulated HAS2 induces PD-L1 expression and suppresses CD8^+^ T cell cytotoxic function [60]. In acute myeloid leukemia, STAT5 transcriptionally activates glycolysis-related genes (HK1, PFKP, PDHA) to promote lactate accumulation. Elevated lactate enhances H4K5la enrichment at the PD-L1 promoter region by promoting E3BP nuclear translocation, thereby driving PD-L1 transcription. Acute myeloid leukemia (AML) cells with high PD-L1 expression significantly suppress CD8^+^ T cell activation in co-culture systems, and PD-1 blockade antibodies can restore T cell function [68]. The pro-glycolytic function of STAT5 is not achieved through direct binding to the PD-L1 promoter but rather through lactate as a metabolic intermediate to indirectly regulate the epigenetic status of PD-L1, revealing a functional link among oncogenes, metabolic reprogramming, and immune checkpoints.

At the protein stability regulation level, lactylation stabilizes PD-L1 protein through competitive inhibition of E3 ubiquitin ligase-mediated degradation, which is a mechanism that is distinct from transcriptional regulation and operates in parallel. In hepatocellular carcinoma, H3K18la-driven major vault protein (MVP) transcriptional upregulation is a key upstream event. MVP, through its cap-helix domain (Ala622-Lys674), competitively binds to β-TrCP, blocking β-TrCP-mediated ubiquitin-proteasome degradation of PD-L1, thereby prolonging PD-L1 protein half-life and enhancing its surface expression. Importantly, MVP regulation does not involve changes in CD274 mRNA levels but operates entirely at the protein degradation level, forming a stable dual-layer regulation together with STAT5-H3K18la-driven PD-L1 transcriptional activation. Notably, the S176A mutation of PD-L1 can eliminate β-TrCP-mediated degradation, indicating that phosphorylation at this site is a prerequisite for β-TrCP recognition, and MVP blocks this recognition process through steric hindrance. At the clinical level, among hepatocellular carcinoma (HCC) patients receiving anti-PD-1/PD-L1 therapy, MVP expression levels in the disease progression group were significantly higher than those in the partial response group, and high MVP expression correlated with shorter overall survival, suggesting that MVP may serve as a potential biomarker for predicting immunotherapy resistance [69].

Beyond PD-L1, the involvement of lactylation in regulating other immune checkpoint molecules remains in early exploratory stages. TIM-3, an important marker of T cell exhaustion, synergizes with PD-1 to promote T cell dysfunction in various tumors [70,71]. However, direct evidence for lactylation-mediated regulation of TIM-3 or LAG-3 expression is currently lacking. In a bovine leukemia virus-infected bovine model, TIM-3 expression is upregulated in CD4^+^, CD8^+^, and γδTCR^+^ T cells, and TIM-3 antibody combined with PD-L1 antibody synergistically enhances T cell activation and cytokine production [72], suggesting that TIM-3 expression or function may be influenced by the metabolic microenvironment, though its direct association with lactylation remains to be established. In melanoma, co-expression of LAG-3 and TIM-3 correlates with poorer overall survival [73], but the upstream metabolic signals driving their co-expression remain unclear. Additionally, PD-1 expression on CD8^+^ T cells is also regulated by the lactate environment. Under high-lactate conditions, the proportion of PD-1^+^TIM-3^+^CD8^+^ T cells is significantly elevated, with severely impaired function [71]. However, whether this effect is mediated through direct lactylation of PD-1 regulatory elements awaits experimental verification.

### 4.2. Lactylation Regulates Tumor-Associated Macrophages (TAMs)

Tumor-associated macrophages (TAMs) are among the most abundant immune cells in the tumor microenvironment, and their functional polarization state is closely associated with tumor progression. In most solid tumors, TAMs exhibit an M2-like immunosuppressive phenotype, suppressing antitumor immune responses through secretion of IL-10, TGF-β, and arginase-1. A substantial body of evidence has established lactylation as a central regulator of TAM polarization from both histone-level and non-histone-level mechanisms.

At the histone lactylation level, H3K18la has been established as an important epigenetic mark driving TAM polarization toward the M2 phenotype. During the late phase of M1 macrophage polarization, glycolysis-derived lactate selectively activates the expression of repair-associated genes, including Arg1, through p300-mediated H3K18la modification, promoting macrophage transition from a pro-inflammatory to a tissue-repair state [7]. MCT4 deficiency drives macrophage conversion toward the M2 phenotype by enhancing p300-mediated H3K18la enrichment at the promoter regions of repair genes, including IL-10 and PDHA1 [74]. In ovarian cancer, lactate enhances H3K18la enrichment at the CCL18 promoter region through GPR132 signaling, upregulating CCL18 expression and promoting macrophage-mediated immunosuppression in the tumor microenvironment [75]. In hepatocellular carcinoma, LDHi or MCT1/MCT4 blockade reduces lactate levels and H3K18la modification, thereby inhibiting M2 polarization of TAMs and promoting conversion toward the M1 phenotype [69]. These findings collectively establish H3K18la as an important epigenetic switch for the immunosuppressive polarization of TAMs.

At the non-histone lactylation level, lactylation of METTL3 provides a mechanistically distinct but functionally complementary perspective on TAM immunosuppressive function. In colorectal cancer, H3K18la-driven METTL3 transcriptional upregulation enhances global m6A modification levels in tumor-infiltrating myeloid cells, and lactylation of the METTL3 protein itself further enhances its RNA binding and methyltransferase activity in a positive feedback manner. METTL3-mediated m6A modification promotes STAT3 phosphorylation through stabilization of JAK1 mRNA, enhancing the immunosuppressive function of myeloid cells [76]. In gastric cancer, H3K18la similarly enhances m6A modification through upregulation of METTL3 expression, and METTL3-mediated m6A modification of CCT2 promotes CCT2 protein expression, which in turn suppresses T cell activity by inhibiting Ca^2+^ influx in CD8^+^ T cells [44]. These studies position METTL3-related lactylation as a bridging node between metabolism and the epitranscriptome in TAM immunosuppressive function, a concept that is mechanistically compelling, though still awaiting further validation in additional tumor types.

Tumor-derived lactate is the primary metabolic source driving TAM lactylation. In glioblastoma, TAMs secrete lactate through MCT4, which is taken up by GSCs via MCT1 and induces KU70 K317 lactylation, suppressing cGAS-STING-mediated type I interferon signaling and thereby inhibiting CD8^+^ T cell infiltration and function. A myeloid-specific knockout of Slc16a3 (MCT4) significantly prolongs survival and increases CD8^+^ T cell infiltration in tumor-bearing mice [77]. In hepatocellular carcinoma, LDHi not only reduces lactylation levels in tumor cells themselves but also remodels TAM polarization status, significantly increasing the proportion of tumor-infiltrating CD8^+^ T cells and their effector molecule expression [69]. These findings establish a bidirectional relationship in which TAMs are both producers and recipients of lactate signals, creating a self-reinforcing immunosuppressive circuit that is therapeutically targetable.

### 4.3. Lactylation Regulates T Cell Function

T cells are the core executors of antitumor immunity, and their functional state is profoundly influenced by the metabolic microenvironment. Lactate, as one of the most abundant metabolites in the tumor microenvironment, exhibits complex dose-dependent and cell type-specific regulatory effects on T cells. The evidence base for lactylation in T cell regulation, however, varies considerably across T cell subsets and differentiation states. The roles in CD8^+^ T cell activation and exhaustion are supported by multiple independent studies, whereas findings in regulatory T cells are more heterogeneous and include both well-established and emerging observations.

In CD8^+^ T cells, the functional output of lactylation is closely associated with the differentiation state of the cells. In acutely activated effector CD8^+^ T cells, glycolysis-derived lactate participates in the expression regulation of effector molecules, including granzyme B, through H3K18la and H3K9la modifications. Inhibition of lactyltransferase activity reduces H3K18la and H3K9la levels and impairs CD8^+^ T cell cytotoxicity, whereas the HDAC inhibitor MS275 enhances T cell effector function and suppresses tumor growth by increasing H3K18la and H3K9la levels [78]. Chromatin immunoprecipitation sequencing analysis revealed that H3K9la and H3K18la in activated CD8^+^ T cells are enriched at the promoter and enhancer regions of activation- and effector-related genes, including Cd28 and Gzm, and co-localize with active transcriptional marks, including H3K4me3 and H3K9ac, suggesting that lactylation promotes gene expression through synergy with other histone modifications [79]. H3K9la is simultaneously enriched at genes related to mitochondrial oxidative phosphorylation and fusion, including Opa1, whereas H3K18la is enriched at genes related to glycolysis and mitochondrial fission, suggesting that these two lactylation marks have distinct functional orientations in regulating CD8^+^ T cell metabolic adaptation. In tissue-resident memory CD8^+^ T cells, the maintenance of H3K9la depends on mitochondrial metabolism (fatty acid oxidation and electron transport chain), contrasting sharply with the glycolysis-driven H3K18la mechanism in activated CD8^+^ T cells [78].

In tumor-infiltrating exhausted CD8^+^ T cells, lactylation regulation presents a more complex picture. A well-established mechanism involves the lactate-H3K9la-IL-11-JAK2/STAT3 axis. In head and neck squamous cell carcinoma, tumor cell-derived lactate activates IL-11 transcription through H3K9la enrichment at the IL11 promoter region. IL-11 upregulates the expression of immune checkpoints, including PD-1, TIGIT, CTLA-4, and TIM-3, on CD8^+^ T cell surfaces through the JAK2/STAT3 signaling pathway, while simultaneously suppressing IFN-γ and granzyme B production, leading to CD8^+^ T cell exhaustion [80]. IL-11-specific knockout restores CD8^+^ T cell killing function and enhances anti-PD-1 therapy efficacy [80]. This finding reveals a non-cell-autonomous mechanism by which tumor cells remotely regulate CD8^+^ T cell function through the lactate-H3K9la-IL-11 axis, a pathway that has been validated by both genetic and pharmacological interventions. In melanoma, MCT11 is specifically highly expressed in terminally exhausted CD8^+^ T cells, and its expression is driven by both chronic TCR stimulation and hypoxia (via HIF-1α). MCT11-mediated lactate uptake promotes dysfunction of exhausted T cells, and T cell-specific MCT11 deletion improves effector function and synergizes with anti-PD-1 therapy. MCT11 monoclonal antibody treatment delays tumor growth and induces complete responses in multiple tumor models, with its efficacy depending on a lactate-rich tumor microenvironment [81]. The regulation of CD8^+^ T cells by lactate is not purely inhibitory; appropriate lactate levels can maintain T cell stemness through metabolic support, suggesting that lactate concentration has a functionally optimal range.

In regulatory T cells (Tregs), lactylation-related mechanisms have been explored through both well-established and emerging pathways. A mechanistically well-characterized pathway involves MOESIN K72 lactylation. Lactate enhances TGF-β signaling and FOXP3 stability in Tregs through MOESIN K72 lactylation, which strengthens the interaction between MOESIN and TGF-β receptor I via an additional hydrogen bond, thereby reinforcing Treg immunosuppressive function [38]. This mechanism has been validated by site-specific antibody blockade and functional suppression assays, establishing MOESIN K72la as a bona fide regulatory node in Treg biology.

An emerging observation in Treg biology involves CD38-mediated metabolic adaptation. A recent study by Sarkar et al. (2025) reported that CD38 depletes intracellular NAD^+^ through its NADase activity, limiting mitochondrial TCA cycle flux and reducing α-ketoglutarate (α-KG) production. Low α-KG levels maintain a hypomethylated state at the CNS regions of the Foxp3 locus, ensuring Treg lineage stability and suppressive function. In the lactate-rich tumor microenvironment, where lactate serves as the primary carbon source, CD38^+^ Tregs preferentially direct lactate-derived pyruvate toward phosphoenolpyruvate through pyruvate carboxylase rather than the TCA cycle. This metabolic diversion depends on CD38-mediated NAD^+^ depletion. Supplementation with the NAD^+^ precursor NMN reverses this metabolic adaptation, increasing α-KG and promoting Foxp3 methylation, thereby weakening Treg suppressive capacity. Pharmacological inhibition of CD38 selectively reduces tumor-infiltrating Treg numbers and enhances antitumor immunity [82]. Although this finding awaits independent validation, it provides a compelling link between lactate metabolism, NAD^+^ homeostasis, and Treg epigenetic regulation that warrants further investigation.

### 4.4. Lactylation Regulates Other Immune Cells

Beyond T cells and tumor-associated macrophages, the lactylation regulation of myeloid-derived suppressor cells, dendritic cells, and natural killer cells also constitutes an important dimension of tumor immune microenvironment remodeling. These cell types perform differentiated functions in the tumor immune network, including antigen presentation, innate killing, and immunosuppression. The evidence base for lactylation in these lineages, however, is considerably more heterogeneous than for TAMs or CD8^+^ T cells. The role in MDSCs is supported by multiple independent studies, whereas findings in DCs are largely indirect, and observations in NK cells derive from a single recent report.

Myeloid-derived suppressor cells (MDSCs) are key executors of the immunosuppressive network in the tumor microenvironment. Tumor-derived lactate upregulates TET2 expression through histone H3K18la modification. TET2, as a DNA demethylase, binds to the Arg1 promoter region via STAT3 as a bridge and mediates its demethylation, thereby enhancing Arg1 transcription and enzymatic activity, ultimately reinforcing MDSC-mediated suppression of CD8^+^ T cell proliferation [83]. This study also excluded interference from acidic pH, confirming that lactate regulation of TET2 is substrate-specific. In another independent study, lactate upregulates SGK1 expression through TET2-mediated demethylation of its promoter region, thereby enhancing MDSC glycolytic activity and the release of immunosuppressive molecules (Arg-1, iNOS, ROS). TET2 silencing significantly impairs MDSC suppressive function and delays tumor growth in tumor-bearing mice [84]. Histone lactylation also induces tumor cell secretion of CCL2 and CCL7 through the HCAR1-14-3-3-STAT3 axis, recruiting CCR2^+^ polymorphonuclear MDSCs into the tumor microenvironment and constructing an immunosuppressive network. The FDA-approved drug reserpine, as an HCAR1 inhibitor, reduces PMN-MDSC infiltration and enhances anti-PD-1 efficacy [85]. Collectively, these findings from three independent studies converge on a coherent epigenetic cascade, which is from histone lactylation to TET2 upregulation to target gene demethylation, that converts extracellular lactate signals into sustained MDSC-mediated immunosuppression, though the relative contribution of this pathway across different tumor types remains to be systematically evaluated.

In dendritic cells (DCs), lactylation primarily impairs their antigen presentation function. Tumor-derived lactate inhibits monocyte differentiation into DCs, downregulates CD1a expression, reduces IL-12 secretion, and induces IL-10 production, conferring a tolerogenic phenotype on DCs [86]. In melanoma models, lactate drives conventional DC differentiation toward mature regulatory DCs through the SREBP2 signaling axis. These regulatory DCs suppress CD8^+^ T cell activity and normal DC cross-presentation capacity while promoting Treg differentiation, constructing an immunosuppressive microenvironment [87]. In plasmacytoid DCs, lactate enhances tryptophan metabolism and kynurenine production and suppresses IFN-α secretion through the GPR81 receptor [85]. However, it is important to note that most of these studies have focused on the effects of lactate itself rather than on lactylation specifically, and the direct target genes of histone lactylation in DCs have not been systematically identified. Whether the observed phenotypic changes are mediated through lactylation-dependent epigenetic remodeling or through lactate signaling via its receptors remains an open question.

In natural killer (NK) cells, direct evidence for lactylation-mediated suppression of cytotoxicity has been obtained. High concentrations of lactate in the tumor microenvironment induce extensive lysine lactylation modifications in NK cells, which are closely associated with NAD^+^ metabolic disturbance and mitochondrial fragmentation. Mechanistically, lactylation modifies ROCK1, which in turn regulates DRP1 phosphorylation, inducing mitochondrial hyperfragmentation and ultimately impairing NK cell killing function. Combination therapy with the NAD^+^ precursor nicotinamide riboside and the SIRT3 activator honokiol restores NK cell antitumor activity through activation of the delactylase SIRT3 [88]. In glioblastoma, lactate drives KU70 K317 lactylation in GSCs through the MCT4-MCT1 transport axis, suppressing cGAS-STING-mediated type I interferon signaling and thereby inhibiting NK cell and CD8^+^ T cell infiltration and function [77]. Although these findings collectively suggest that lactylation serves as a functional metabolic checkpoint for NK cells, the field is still nascent. The ROCK1-DRP1 axis in NK cells has been reported in only one study, and the relative contribution of direct lactylation of NK cell proteins versus indirect effects via the tumor microenvironment remains to be disentangled. Independent validation across multiple tumor models and NK cell contexts will be essential to establish this as a generalizable mechanism.

Beyond the conventional NK cells discussed above, lactate also exerts regulatory effects on other members of the innate lymphoid cell (ILC) family. ILC2s, activated by IL-33 through the IL1RL1 (ST2) receptor, contribute to antitumor responses primarily through IL-5-mediated eosinophil recruitment and activation, forming the IL-33/ILC2/eosinophil axis that suppresses melanoma growth. Tumor-derived lactic acid, however, profoundly inhibits ILC2 proliferation and cytokine production (including IL-5 and IL-13) in a dose-dependent manner, with the inhibitory effect being mediated largely through acidification rather than lactate itself, as neutralization of pH or the use of sodium lactate abolishes this suppression. Consistently, melanomas with reduced LDHA expression and consequently lower lactate production exhibit increased intratumoral ILC2 infiltration and enhanced responsiveness to IL-33 therapy compared to control tumors. Clinical correlative data further support this link: in human cutaneous melanoma, LDHA expression negatively correlates with ILC2-associated markers including KLRG1, GATA3, and ICOS, whereas high IL33 expression is associated with better overall survival [89,90].

Lactate also modulates other ILC subsets. A recent study by Lim et al. (2023) demonstrated that tumor-derived lactate enhances PD-1 expression on T-bet^+^NK1.1^−^ ILCs (an ILC1-like subset) within the tumor microenvironment, leading to dampened mTOR signaling and increased fatty acid uptake, which collectively suppress ILC effector function. PD-1 blockade reversed these metabolic alterations and restored IFN-γ and granzyme B/K production, thereby enhancing antitumor immunity in murine melanoma models [91]. These findings firmly establish lactate as a negative regulator of both ILC2s and ILC1-like subsets. However, direct evidence for lysine lactylation in ILCs is currently absent, rather than lactate signaling via pH changes, MCT-mediated transport, or receptor engagement. Whether lactylation directly modifies key ILC transcription factors (such as GATA3 in ILC2s) or metabolic regulators to mediate these immunosuppressive effects remains entirely unexplored. The regulatory role of lactate in ILC biology is primarily documented through metabolic and pH-dependent pathways, whereas the contribution of protein lactylation to ILC function represents a largely uncharted territory. Nonetheless, the emerging ILC–lactate axis offers a promising target for combinatorial immunotherapy, particularly in melanoma and other skin cancers where ILCs are abundant [90].

### 4.5. Lactylation Regulates Antigen Presentation

Antigen presentation is a core link connecting tumor antigen recognition to T cell effector activation, and its efficiency directly determines whether CD8^+^ T cells can effectively recognize and eliminate tumor cells. Evidence linking lactylation to antigen presentation, however, is currently preliminary and largely indirect, with most studies reporting correlative observations rather than mechanistic dissections of antigen processing or MHC loading pathways. Lactylation, as a key modification bridging metabolic reprogramming and epigenetic regulation, has been found in recent years to impair the integrity of antigen presentation pathways at multiple levels, thereby weakening antitumor immune responses.

At the molecular level, lactylation can regulate the expression of antigen presentation-related genes through both histone and non-histone pathways. In non-small cell lung cancer, H3K18la enrichment at the POM121 promoter region promotes POM121 transcription. POM121 enhances PD-L1 expression by promoting MYC nuclear translocation, indirectly impairing T cell recognition efficiency of tumor antigens [74]. In head and neck squamous cell carcinoma, H3K9la activates IL-11 transcription, which upregulates immune checkpoints including PD-1, TIGIT, CTLA-4, and TIM-3 on CD8^+^ T cell surfaces through the JAK2/STAT3 signaling pathway, inhibiting T cell antigen response capacity [80]. These findings indicate that lactylation indirectly interferes with the T cell effector phase following antigen presentation through upregulation of immune checkpoint molecules.

Lactylation and O-GlcNAc modification may exhibit functional crosstalk in antigen presentation regulation. O-GlcNAc transferase (OGT)-mediated O-GlcNAc modification at TAP1 Ser63 in bladder cancer disrupts the interaction between TAP1 and HLA-A, preventing MHC-I molecules from effectively loading antigenic peptides and translocating to the cell membrane. Instead, they are degraded through the autophagy pathway, ultimately impairing CD8^+^ T cell-mediated tumor cell killing [92]. Although this study focused on O-GlcNAc modification rather than lactylation, given that both O-GlcNAc and lactylation are regulated by glycolytic flux and may compete for the same lysine residues on proteins, lactylation may directly modify TAP1 or MHC-I molecules themselves through analogous mechanisms. However, direct experimental evidence for such a model is currently absent. In non-alcoholic steatohepatitis, tRF-31R9J reduces H3K18la modification levels at the promoter regions of ATF3, ATF4, and CHAC1 by recruiting HDAC1, suppressing the transcription of these pro-ferroptotic genes [57], suggesting that lactylation may indirectly influence the expression profile of antigen presentation-related genes through epigenetic regulation.

In the broader immune regulatory network, preliminary evidence for lactylation-mediated suppression of dendritic cell antigen presentation function has been obtained. Tumor-derived lactate inhibits monocyte differentiation into DCs, downregulates CD1a expression, reduces IL-12 secretion, and confers a tolerogenic phenotype on DCs [86]. In melanoma models, lactate drives conventional DC differentiation toward mature regulatory DCs through the SREBP2 signaling axis, suppressing their cross-presentation capacity and promoting Treg differentiation [87]. These effects may involve histone lactylation-mediated transcriptional regulation of antigen processing and presentation-related genes (including TAP1, PSMB9, and MHC-I) in DCs, but direct evidence remains insufficient at present.

In summary, despite the plausible conceptual framework linking lactylation to antigen presentation, the field remains in its infancy. The critical distinction between lactate signaling via its receptors versus lactate as a substrate for lactylation has not been systematically addressed in this context. Direct evidence for the lactylation of MHC-I, TAP1, or other components of the antigen processing machinery is entirely absent, and the majority of observations are indirect, derived from checkpoint upregulation or non-tumor contexts. Until these gaps are filled by targeted mechanistic studies, ideally involving site-specific lactylation mapping of antigen presentation components and functional assays of peptide loading and TCR engagement, the role of lactylation in antigen presentation should be considered a promising but speculative area of investigation.

## 5. Therapeutic Implications and Clinical Translation of Lactylation

### 5.1. Diagnostic and Prognostic Biomarkers

The aberrant elevation of lactate levels in tumor tissues and the consequent lysine lactylation modifications provide novel molecular dimensions for early diagnosis, prognosis assessment, and prediction of therapeutic response. In recent years, risk scoring models constructed based on lactylation-related gene expression profiles have demonstrated independent prognostic predictive value across multiple malignancies, forming a multi-layered biomarker system spanning metabolite concentrations, modification levels, and gene signatures. It is important to note, however, that most current evidence for lactylation-based biomarkers derives from retrospective analyses, and their clinical utility awaits confirmation in prospective multi-center cohorts.

At the prognostic assessment level, multiple independent research teams have constructed lactylation-related gene signatures using public databases and validated their significant associations with patient survival. Huang et al. (2024) integrated single-cell and bulk transcriptomic analyses to identify 23 lactylation-related core genes in colorectal cancer. The risk model successfully stratified patients into high-risk and low-risk groups in both the TCGA cohort and two independent gene expression omnibus (GEO) validation sets, with high-risk patients exhibiting significantly shorter overall survival. This study further validated the high protein-level expression of risk genes, including AP2M1, TERF2IP, and ARL4C, in tumor tissues through tissue microarray immunohistochemistry, confirming the clinical translatability of lactylation-related gene signatures [93]. In breast cancer, Sun et al. (2025) screened lactylation-related metastasis-associated differentially expressed genes from the TCGA database and constructed an 8-gene risk model, including GBP2, SLC5A8, STAT4, and CLEC2D. This model not only demonstrated robust prognostic stratification in the training cohort but also showed that high-risk patients in three external validation sets (GSE11121, GSE6532, and GSE19615) had significantly worse distant relapse-free survival and metastasis-free survival than low-risk patients. The study further confirmed through immunohistochemistry and western blot that global lactylation levels in metastatic lesions were significantly higher than in primary lesions, directly validating the association between lactylation and tumor progression at the protein modification level [94].

In biomarker studies of specific cancer types, Wu et al. (2026) focused on the antioxidant protein PRDX1 and identified it as a key lactylation-related gene in breast cancer through Mendelian randomization analysis. Single-cell sequencing revealed that PRDX1 is significantly expressed in monocytes, with notable intercellular communication between PRDX1-positive monocytes and fibroblasts. A 9-gene prognostic model constructed based on PRDX1-positive monocyte differentially expressed genes demonstrated good 1-, 3-, and 5-year survival prediction performance in both the TCGA training cohort and GEO validation sets (AUC values of 0.681, 0.748, and 0.700, respectively). Spatial transcriptomics further revealed the spatial heterogeneity of PRDX1 expression, with high expression signals concentrated in tumor nests rather than stromal regions, suggesting that the spatial distribution characteristics of lactylation-related markers may be closely associated with the functional status of the local tumor microenvironment [95]. Zhou et al. (2026) employed an integrated machine learning strategy combining SVM-RFE and LASSO-Cox regression in lung adenocarcinoma, screening four key genes from 446 lactylation-related genes: CCNA2, PRAM1, GPR37, and HMGA1. The risk model achieved 1-, 3-, and 5-year AUC values of 0.675, 0.747, and 0.742 in time-dependent ROC analysis. The innovation of this study lies in incorporating single-cell sequencing data into the analytical pipeline, revealing the differential expression of HMGA1 and PRAM1 in macrophages, monocytes, and fibroblasts, suggesting that lactylation-related gene signatures may influence patient prognosis by remodeling the metabolic and functional states of immune cells [96].

In hepatocellular carcinoma, prognostic models based on lactylation-related genes have also demonstrated clinical application potential. Zou et al. (2026) evaluated the expression and prognostic value of AARS2 in colon adenocarcinoma and other malignancies through a Pan-Cancer Analysis of Whole Genomes. Using a multi-algorithm consensus strategy, AARS2 was identified as a core prognostic gene among 160 lactylation-related genes. Single-cell and spatial transcriptomic analyses both confirmed that AARS2 is primarily enriched in malignant cell populations. In the TCGA-COAD cohort, patients with high AARS2 expression had significantly worse overall survival, progression-free interval, and disease-specific survival than those with low expression. Multivariate Cox regression confirmed AARS2 expression, T stage, and overall stage as independent prognostic factors. The study further validated high AARS2 protein expression in tumor tissues through immunohistochemistry in independent clinical samples, providing protein-level evidence for clinical detection of lactylation-related genes [97].

The prognostic value of lactylation-related gene signatures demonstrates certain consistency across cancer types while also exhibiting lineage-specific differences. Yang et al. (2025) focused on radioresistant non-small cell lung cancer and identified three lactylation-related radioresistance genes, RRM2B, COL4A1, and CD46, by intersecting differentially expressed genes from radioresistant cell lines with lactylation gene sets. The risk model showed robust prognostic stratification in the TCGA-LUAD radiotherapy patient cohort, with 1-, 3-, and 5-year disease-free survival AUC values reaching 0.76, 0.847, and 0.89, respectively. Notably, the study correlated lactylation risk scores with tumor microenvironment immune features. High-risk patients exhibited significant enrichment of M2 macrophages, myeloid-derived suppressor cells, and regulatory T cells, accompanied by suppression of CD8^+^ T cell function, suggesting that lactylation-related gene signatures may drive therapeutic resistance and poor prognosis by shaping an immunosuppressive microenvironment [98].

The diagnostic or prognostic capacity of lactylation-related gene signatures does not arise from the independent action of single genes but rather reflects systemic dysregulation of the lactylation modification network in tumor cells and the microenvironment. From the biomarker application perspective, lactylation-related gene signatures offer higher information dimensionality and more robust predictive performance compared to single metabolites or single modification sites. However, the construction of most current models still relies on retrospective public databases, and their generalizability across different populations and treatment contexts requires prospective cohort validation. Furthermore, the dynamic reversibility of lactylation modification implies that static gene expression signatures may not fully capture real-time changes in the lactylation landscape during treatment. Integrating lactylation gene signatures with modificomics monitoring in liquid biopsy may represent the next direction for biomarker development.

### 5.2. Targeting Lactate Metabolism to Suppress Lactylation

**Figure 4:**
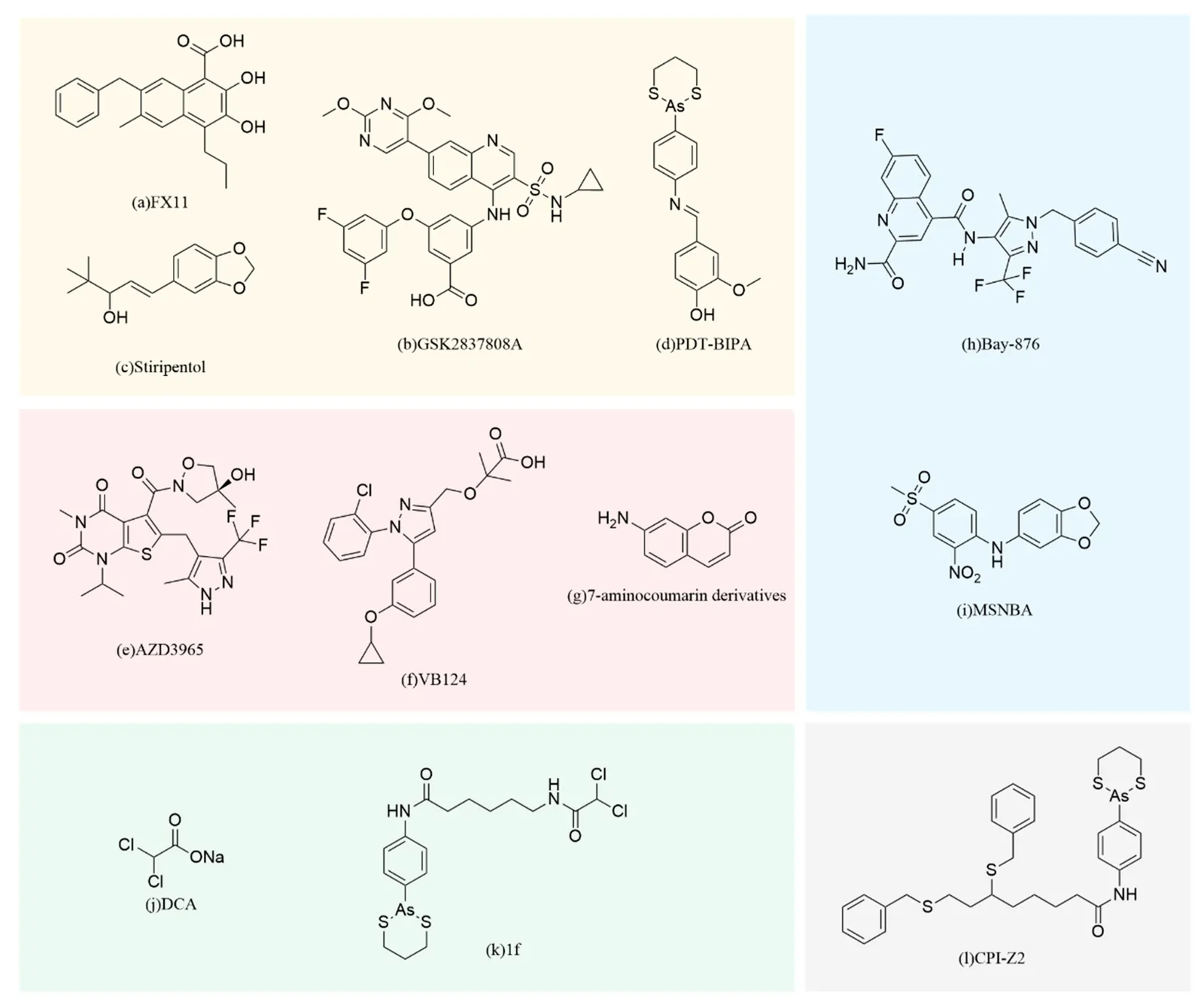
This figure illustrates the chemical structures of inhibitors in the lactate metabolic pathways. (**a**–**d**) LDHA inhibitors. (**e**–**g**) MCT inhibitors. (**h**,**i**) GLUT inhibitors. (**j**,**k**) PDK1 inhibitors. (**l**) PDHC/KGDHC dual-targeting inhibitor CPI-Z2.

Given that lactate serves as the direct substrate for lactylation, its intracellular concentration constitutes the core determinant of lactylation levels. Targeting key nodes in the lactate metabolic pathway to reduce lactate production or block lactate transport has been extensively explored in preclinical models as an important strategy for suppressing lactylation and interfering with malignant tumor progression. This direction encompasses multiple druggable targets, including lactate dehydrogenase, monocarboxylate transporters, glucose transporters, and pyruvate dehydrogenase kinase, with intervention strategies at each target demonstrating varying degrees of antitumor potential in preclinical and early clinical studies (Table 3). The clinical translation of these metabolic interventions, however, is still in its infancy, and multiple obstacles, including tumor metabolic plasticity and compensatory transporter upregulation, have become apparent even in preclinical settings. Figure 4 shows the chemical structures of representative inhibitors targeting key enzymes and transporters in the lactate metabolic pathway (Figure 4).

Lactate dehydrogenase (LDH) is the terminal enzyme of the glycolytic pathway, catalyzing the reductive conversion of pyruvate to lactate. LDHA is highly expressed in multiple solid tumors, and its activity directly determines the size of the intracellular lactate pool and the supply of lactylation substrates [102]. The LDHA inhibitor FX11 significantly reduces tumor lactate levels and inhibits growth in non-small cell lung cancer and pancreatic cancer xenograft models. Furthermore, the combination of FX11 with anti-PD-1 therapy demonstrated synergistic antitumor efficacy in a humanized mouse model of non-small cell lung cancer, with mechanisms involving increased CD8^+^ T cell infiltration and restoration of effector function [109]. Another LDHA inhibitor, GSK2837808A, enhanced the efficacy of adoptive T cell therapy in melanoma models, further validating the feasibility of integrating metabolic regulation with immunotherapy [98]. In glioblastoma, the LDHA inhibitor Stiripentol significantly enhanced temozolomide chemosensitivity by reducing lactate-mediated H3K9 lactylation levels, with its blood–brain barrier penetration offering a unique advantage for brain tumor therapy [110]. Liu et al. (2019) reported that the organic arsenical PDT-BIPA directly inhibits LDHA activity, shifting the energy metabolism of tumor cells from glycolysis to oxidative phosphorylation. This shift triggers a burst of reactive oxygen species and mitochondrial dysfunction, subsequently inducing caspase family-dependent apoptosis. In a leukemia-bearing mouse model, PDT-BIPA achieved a tumor inhibition rate exceeding 60% at a dose of 1.6 mg/kg, with no significant hepatorenal toxicity or body weight loss observed. These findings demonstrate the therapeutic potential and safety window of LDHA-targeted inhibition in vivo [111]. However, LDHA single-target inhibition faces the challenge of tumor metabolic plasticity. Long-term LDHA inhibition can trigger compensatory upregulation of LDHB, forcing tumor cells to shift toward lactate oxidation pathways for energy maintenance [104]. This finding suggests that dual intervention strategies simultaneously targeting LDHA and LDHB may achieve superior therapeutic outcomes.

The MCT mediates transmembrane transport of lactate across cell membranes, with MCT1 and MCT4 playing complementary roles in maintaining lactate homeostasis in tumor cells. MCT1 is primarily responsible for lactate uptake, whereas MCT4 mediates lactate efflux from glycolytically active tumor cells, preventing excessive intracellular lactate accumulation [105]. The MCT1-selective inhibitor AZD3965 is currently the most advanced lactate transport-targeting drug, having completed Phase I/II clinical trials in patients with advanced solid tumors and diffuse large B-cell lymphoma (NCT01791595). This study established the tolerated dose and pharmacokinetic profile of AZD3965, with dose-limiting toxicity primarily consisting of reversible ocular changes, suggesting a favorable safety window for MCT1 targeting. In preclinical models, AZD3965 reduced tumor cell lactate release and remodeled the immune microenvironment, enhancing the efficacy of anti-PD-1 immunotherapy. The MCT4 inhibitor VB124 enhanced CD8^+^ T cell infiltration and cytotoxic function in hepatocellular carcinoma models, with combination therapy with anti-PD-1 significantly prolonging survival in tumor-bearing mice. Given the functional compensation between MCT1 and MCT4, 7-aminocoumarin derivatives achieve dual blockade by simultaneously inhibiting both MCT1 and MCT4, effectively preventing compensatory lactate transport following single-target inhibition [14,106].

Glucose transporter 1 (GLUT1) inhibitors reduce glycolytic flux and lactate production at the source by limiting glucose uptake. The GLUT1-selective inhibitor BAY-876 has demonstrated antiproliferative activity in multiple solid tumor models, with mechanisms involving a dual restriction of energy crisis due to glucose deprivation and lactylation substrate supply [107]. However, functional redundancy among GLUT family members and the physiological glucose requirements of normal tissues impose high demands on the selectivity of this target. GLUT5, a fructose-specific transporter aberrantly overexpressed in certain tumors, provides a pathway for tumor cells to utilize fructose as an alternative carbon source under glucose-limited conditions. Its inhibitor MSNBA has shown selective antiproliferative effects in colon cancer cells [108].

Pyruvate dehydrogenase kinase 1 (PDK1) inhibits the conversion of pyruvate to acetyl-CoA by phosphorylating the pyruvate dehydrogenase complex, thereby diverting carbon flux from mitochondrial oxidative phosphorylation toward lactate production. The PDK1 inhibitor dichloroacetate activates the PDH complex, promoting preferential entry of pyruvate into the TCA cycle rather than its reduction to lactate, thereby reducing intracellular lactate levels and the extent of lactylation modification. In non-small cell lung cancer models, the combination of dichloroacetate with EGFR tyrosine kinase inhibitors and ionizing radiation significantly enhanced antitumor efficacy, with mechanisms involving microenvironment deacidification and metabolic reprogramming resulting from reduced lactate secretion [112]. She et al. (2023) systematically modified the structure of dichloroacetate and incorporated an organic arsenical pharmacophore to design a series of PDK inhibitors. Among these, the lead compound 1f inhibited PDK1 with an *EC*_50_ as low as 68 nM at the enzymatic level, representing a more than 700-fold improvement over dichloroacetate (*EC*_50_ > 50 μM). In a 4T1 breast cancer xenograft model, 1f (0.15 mg/kg) effectively suppressed PDK activity, activated PDHC, shifted energy metabolism from glycolysis to oxidative phosphorylation, and induced tumor cell apoptosis. When encapsulated in an albumin-hybrid membrane nano-delivery system, the tumor inhibition rate at the same dose increased from 35% to 90% [113]. Dichloroacetate has also demonstrated sensitizing effects in CAR-T cell therapy, suggesting its potential for combination with cellular Immunotherapies.

Targeting lactate metabolism encompasses a complete pathway from substrate supply to transport regulation, with inhibition strategies at different targets emphasizing distinct aspects of reducing lactate levels, remodeling the tumor microenvironment, and sensitizing immunotherapy. However, tumor metabolic plasticity and the presence of compensatory pathways render single-target interventions often insufficient for achieving durable efficacy. She et al. (2024) further constructed a nanoparticle system, AMANC@M, capable of sequentially releasing a CRISPR/Cas9 gene-editing system and CPI-Z2, a dual-targeting inhibitor of PDHC and KGDHC. This nanoparticle simultaneously suppressed glycolysis through CRISPR/Cas9-mediated LDHA knockdown and blocked the tricarboxylic acid cycle through CPI-Z2 release, achieving dual metabolic inhibition of both glycolysis and oxidative phosphorylation. In a 4T1 triple-negative breast cancer xenograft model, a single injection of AMANC@M achieved a tumor inhibition rate of 90% and effectively reversed the hypoxic and acidic states of the tumor microenvironment, promoting the infiltration of CD4^+^ and CD8^+^ T cells [114]. This study provides a nanotechnology platform for multi-target synergistic intervention targeting the upstream regulators of lactate metabolism, PDK and LDHA. Therefore, multi-target combination interventions targeting the lactate metabolic network, along with rational combinations with immune checkpoint blockade, chemotherapy, or targeted therapy, will be key directions for clinical translation in this domain.

### 5.3. Targeting Lactyltransferases and Delactylases

The dynamic equilibrium of lactylation is maintained by lactyltransferases that catalyze the addition of lactyl groups and delactylases that catalyze their removal. Targeting lactyltransferases to inhibit lactyl group deposition or targeting delactylases to accelerate lactyl group removal constitutes complementary strategies for modulating lactylation levels. Unlike indirect regulation through targeting lactate metabolism, direct intervention on the enzymatic machinery theoretically enables more selective modulation of the modification. In recent years, with the progressive elucidation of the enzymatic mechanisms of lactylation, multiple lactyltransferases and delactylases have been proposed as potential druggable targets, among which p300/CBP and their associated inhibitors have been most extensively studied.

p300/CBP were the first identified lactyltransferases, catalyzing the lactylation of histones and non-histone proteins using lactyl-CoA as a donor [7]. In multiple tumor types, p300/CBP overexpression is significantly associated with elevated lactylation levels and poor prognosis. Inhibitors targeting p300/CBP fall into two main categories. The HAT domain inhibitor A-485 is the most extensively studied selective p300/CBP catalytic inhibitor. It effectively inhibits the acetyltransferase activity of p300 and CBP at nanomolar concentrations (*IC*_50_ of 9.8 nM and 2.6 nM, respectively) by competing for the acetyl-CoA binding site [115]. In multiple tumor models, A-485 significantly reduces global and site-specific lactylation levels and suppresses androgen receptor- and MYC-driven transcriptional programs [116]. However, the non-selective inhibition of both lactylation and acetylation by A-485 poses a major obstacle for clinical application. Given that p300/CBP simultaneously catalyze both lactylation and acetylation, the differential inhibition of these two modifications by A-485 makes it difficult to attribute its antitumor effects specifically to reduced lactylation versus altered acetylation [117]. The second category comprises bromodomain inhibitors such as CCS1477 (inobrodib), which disrupt p300/CBP chromatin binding and transcriptional coactivator function by blocking the recognition of acetylated lysines. CCS1477 is currently the most advanced p300/CBP inhibitor, having completed Phase I/IIa clinical trials in metastatic castration-resistant prostate cancer and hematologic malignancies (NCT03568656). Early data indicate that CCS1477 reduces the expression of MYC and androgen receptor target genes in tumor tissues, with treatment responses observed in some acute myeloid leukemia and multiple myeloma patients [99,118]. However, bromodomain inhibitors face similar specificity challenges, with limited capacity to differentiate downstream effects on lactylation versus acetylation.

Activation of delactylases represents another class of intervention strategies. HDAC1-3 and SIRT1-3 have been confirmed to possess delactylase activity, with HDAC1-3 exhibiting efficient removal capacity for both L-lactylation and D-lactylation, and HDAC3 showing the most prominent catalytic efficiency among delactylases [8]. HDAC inhibitors such as vorinostat and entinostat have been clinically used for tumor therapy [119], and their effects on lactylation are increasingly drawing attention. Emerging evidence indicates that HDAC inhibitors can reduce global lactylation levels in tumor cells by enhancing delactylase activity. The dual regulatory effects of HDAC inhibitors on both lactylation and acetylation complicate mechanistic interpretation. In MCF-7 cells, histone acetylation reaches equilibrium within 6 h, whereas lactylation continues to rise over 24 h, suggesting a significant temporal scale difference in the dynamics of these two modifications [7]. Therefore, the relative impact of HDAC inhibitors on lactylation versus acetylation may differ across time windows, a property that represents both a potential therapeutic advantage and a factor requiring careful evaluation in dose and schedule optimization.

SIRT family activators represent another pathway for enhancing delactylation. Honokiol, a natural SIRT3 activator, inhibits cell cycle progression and induces tumor cell apoptosis in hepatocellular carcinoma by enhancing SIRT3-mediated delactylation at CCNE2 K348 [28]. Conversely, the SIRT2 inhibitor AGK2 promotes cuproptosis in gastric cancer cells by blocking METTL16 delactylation and enhancing its lactylation level [25]. This seemingly paradoxical observation indicates that the functional output of lactylation is highly dependent on the identity of the substrate protein and the biological context of the modification site. Therapeutic strategies targeting delactylases require careful evaluation of their directional effects across different tumor types and substrate contexts.

### 5.4. Combination Strategies of Lactylation-Targeted Therapy and Immunotherapy

Given the multi-layered regulatory roles of lactate metabolism and lactylation in the tumor immune microenvironment, combination strategies targeting this metabolic–epigenetic axis with immune checkpoint blockade have attracted considerable preclinical interest. Preclinical studies have systematically evaluated the synergistic antitumor effects of targeting three key nodes—lactate production, lactate transport, and lactylation enzymatic machinery—in combination with PD-1/PD-L1 blockade, providing theoretical foundations and experimental support for subsequent clinical translation. It should be emphasized, however, that virtually all evidence for such combinations currently derives from murine tumor models. Clinical data are scarce, and the optimal timing, dosing, and patient selection for these combinations remain undefined.

Lactate dehydrogenase, as the terminal enzyme of the glycolytic pathway, directly determines the size of the intracellular lactate pool and the supply of lactylation substrates through its catalytic activity. The LDHA inhibitor FX11 has demonstrated synergistic effects with immune checkpoint blockade in non-small cell lung cancer and melanoma models. Qiao et al. (2021) showed that FX11 combined with anti-PD-1 therapy significantly enhanced CD8^+^ T cell infiltration and effector function in a humanized mouse model of non-small cell lung cancer, with mechanisms involving reduced lactate concentrations in the tumor microenvironment and consequent restoration of T cell function [109]. In pancreatic cancer models, genetic silencing of LDHA similarly enhanced the antitumor efficacy of chimeric antigen receptor T cell therapy [120]. The LDHA inhibitor ML-05 in the B16F10 melanoma model further confirmed that this compound not only inhibited tumor growth as a monotherapy but also exhibited enhanced antitumor activity when combined with anti-PD-1 antibody or STING agonists, with effects closely associated with increased proportions of Th1 cells and granzyme B-positive CD8 T cells in the tumor microenvironment [103,121]. However, LDHA single-target inhibition faces the challenge of tumor metabolic plasticity. Long-term LDHA inhibition can trigger compensatory upregulation of LDHB, suggesting that dual intervention strategies simultaneously targeting LDHA and LDHB may achieve superior therapeutic outcomes.

The monocarboxylate transporter family mediates the transmembrane transport of lactate across cell membranes, with MCT1 and MCT4 playing complementary functions in maintaining lactate homeostasis in tumor cells. In preclinical models, the MCT1-selective inhibitor AZD3965 remodeled the immune microenvironment by reducing tumor cell lactate release and enhancing the efficacy of anti-PD-1 immufnotherapy [122]. The MCT4 inhibitor VB124 enhanced CD8^+^ T cell infiltration and cytotoxic function in hepatocellular carcinoma models, with combination therapy with anti-PD-1 significantly prolonging survival in tumor-bearing mice [123]. Given the functional compensation between MCT1 and MCT4, dual blockade strategies simultaneously inhibiting both effectively prevent compensatory lactate transport following single-target inhibition. The regulation of PD-L1 stabilization by lactylation itself provides a new combination target for immune checkpoint blockade. Liang et al. (2026) found that lactate inhibits PD-L1 ubiquitin-proteasome degradation through AARS1-mediated PD-L1 K280 lactylation, thereby stabilizing PD-L1 protein levels. In preclinical models, sodium lactate administration, although not directly affecting tumor growth, significantly enhanced the efficacy of anti-PD-L1 therapy. The mechanism lies in the lactylation-maintained high PD-L1 expression, providing more abundant targets for the antibody. This finding reveals a seemingly paradoxical yet highly translationally valuable strategy: maintaining rather than eliminating PD-L1 expression may optimize immunotherapy efficacy by increasing antibody accessibility [124].

Combination strategies of histone deacetylase inhibitors with immune checkpoint blockade also demonstrate clinical translation prospects. HDAC6, as a key target regulating PD-L1 expression and tumor-associated macrophage polarization, when combined with its selective inhibitor Nexturastat A and anti-PD-1 antibody in melanoma models, significantly inhibited tumor growth, with some mice achieving complete remission [125]. Mechanistic studies indicated that this combination strategy not only reduced PD-L1 expression on tumor cell surfaces but, more importantly, remodeled macrophage polarization status in the tumor microenvironment, increased the M1/M2 ratio, and promoted the infiltration of CD8^+^ T cells and natural killer cells. Zheng et al. (2016) systematically evaluated the capacity of multiple HDAC inhibitors to induce T cell chemokine expression, finding that romidepsin promoted T cell infiltration through the upregulation of CXCL9 and CXCL10 in lung cancer models and induced complete tumor rejection when combined with anti-PD-1. Notably, this study also revealed the critical role of the IFNγ-chemokine positive feedback loop in combination therapy, with the neutralization of IFNγ or blockade of chemokine receptors completely abolishing the antitumor effects of the combination therapy [126]. HDAC5 inhibits PD-L1 transcription through the deacetylation of NF-κB p65 at K310 in pancreatic cancer, and its inhibitor LMK235 combined with anti-PD-1 significantly prolonged the survival of tumor-bearing mice in KPC-derived pancreatic cancer models [127]. The HDAC6 inhibitor ACY-1215 in colorectal cancer models inhibited STAT1 phosphorylation and nuclear translocation by promoting STAT1 acetylation, thereby downregulating PD-L1 expression and enhancing T cell-mediated tumor cell killing [58]. This finding complements the study by Knox et al. (2019) in target selection, suggesting that different HDAC family members exhibit isoform-specific regulation of the tumor immune microenvironment [125].

The design of dual-target inhibitors offers a more streamlined strategy for combination therapy. Yuan et al. (2025) first reported biphenyl hydroxamic acid compounds with both PD-L1 inhibitory and class I HDAC inhibitory activities. Compound 14, while inhibiting HDAC2/3 activity, could upregulate PD-L1 and CXCL10 levels in tumor cells with low PD-L1 expression, improving the immunotherapy response by recruiting T cell infiltration. The design concept of such bifunctional molecules provides new ideas for simplifying combination regimens and reducing drug–drug interaction risks [128]. Zhu et al. (2024) developed a PARP/HDAC dual-target inhibitor that enhanced tumor immunogenicity by inducing BRCAness and cGAS-STING pathway activation in triple-negative breast cancer models, exhibiting synergistic effects with the anti-PD-L1 antibody. Although the clinical translation of these dual-target inhibitors remains in early stages, their design concepts offer a reference paradigm for the combined intervention of lactylation-related targets [129].

From a clinical perspective, the effectiveness of the aforementioned combination strategies depends on the precise identification of patient stratification biomarkers. Serum LDH levels have been established as independent prognostic factors for immunotherapy outcomes in multiple clinical cohorts. In the reanalysis of the IMpower133 study, Shang et al. (2025) found that extensive-stage small cell lung cancer patients with high LDH levels did not derive a survival benefit from atezolizumab therapy, whereas patients with low LDH levels had significantly prolonged median survival. Further analysis revealed that LDH levels negatively correlated with CD8^+^ T cell infiltration and T cell receptor (TCR) clonal diversity, with mechanisms involving lactate upregulating Nur77 expression in naïve CD8^+^ T cells through H3K18la lactylation, inducing tonic TCR signaling and impairing T cell responsiveness to antigenic stimulation. This finding directly links serum LDH, a clinically routine parameter, to lactylation-driven immune evasion mechanisms, providing a theoretical basis for patient selection in combination therapy [130].

Combination strategies targeting the lactylation metabolic–epigenetic axis and immune checkpoint blockade have demonstrated synergistic antitumor effects across multiple preclinical models, with mechanisms encompassing inhibition of lactate production, blockade of lactate transport, regulation of lactylation enzymatic machinery, and intervention in PD-L1 stabilization. The efficacy of different intervention strategies varies by tumor type, microenvironment characteristics, and combination regimens, suggesting that future clinical translation should optimize combination therapy design based on biomarker-guided patient stratification. Clinical trials guided by LDH or lactylation modification levels will provide key evidence for the clinical feasibility of these strategies.

### 5.5. Current Status of Clinical Studies and Translational Challenges

Despite the growing body of preclinical evidence for targeting lactate metabolism and lactylation modifications, translation to clinical application remains in the early exploratory stages. Currently, several intervention strategies targeting the lactate metabolic pathway have entered clinical trial evaluation. The MCT1-selective inhibitor AZD3965 has completed Phase I/II clinical trials in patients with advanced solid tumors and diffuse large B-cell lymphoma (NCT01791595), establishing the tolerated dose and pharmacokinetic profile. The dose-limiting toxicity primarily consisted of reversible ocular changes, suggesting a favorable safety window for this target [131]. The PDHK1 inhibitor dichloroacetate has also been evaluated in multiple clinical trials (NCT00566410), demonstrating enhanced sensitivity to chemoradiotherapy and immunotherapy through reduced lactate secretion in non-small cell lung cancer models [112]. However, these early clinical studies have predominantly focused on safety and tolerability as primary endpoints and have not systematically evaluated the remodeling effects of these interventions on lactylation modification levels and the immune microenvironment in tumor tissues.

As a molecular bridge connecting tumor metabolism and immune regulation, lactylation modification faces multiple challenges in clinical translation. First, standardized detection methods for lactylation modification are lacking. Current assessment of lactylation levels in tissue samples primarily relies on immunohistochemistry or mass spectrometry. The former is limited by antibody specificity, whereas the latter requires high-quality samples and has limited throughput. Liang et al. (2026) developed a PD-L1 K280 site-specific lactylation antibody, providing a tool for detecting specific modification sites in clinical samples. This study validated the association between PD-L1 lactylation and patient prognosis in non-small cell lung cancer tissue microarrays through multiplex immunohistochemistry, offering proof of concept for the feasibility of lactylation modification as a clinical biomarker [124].

Second, lactylation shares enzymatic regulatory networks with other acylation modifications, including acetylation, presenting structural pharmacology challenges for developing highly selective lactyltransferase or delactylase inhibitors. p300/CBP, as both a lactyltransferase and acetyltransferase, inevitably affects the acetylation modification network when its inhibitors block lactylation [17,128]. The discovery of AARS1/2 as novel lactyltransferases provides new targets for developing more selective intervention strategies, but their clinical validation will require time [21,132].

Third, and perhaps most critically, the functions of lactylation differ between tumor cells and immune cells. In tumor cells, lactylation drives malignant progression and immune evasion, whereas in T cells, appropriate lactate levels maintain stem-like phenotypes through lactylation [133]. This duality suggests that systemic lactate suppression or lactylation blockade may produce unintended immunosuppressive effects, and precise definition of the therapeutic window will be an unavoidable issue in clinical translation. Future research directions should focus on developing site-specific lactylation intervention strategies and establishing patient stratification methods based on lactylation modification profiles to facilitate the substantial transition of this field from proof of concept to clinical application.

## 6. Crosstalk Between Lactylation and Other Post-Translational Modifications

### 6.1. Synergy and Antagonism with Acetylation

Lactylation and acetylation, as two of the most prevalent acylation modifications on lysine residues, often share the same chemical reaction sites and partial enzymatic regulatory networks. They form complex synergistic and antagonistic networks at the levels of metabolic substrate supply and enzymatic catalysis competition.

H3K18 represents the most extensively studied case of same-site competition. Zhang et al. (2019) discovered, upon first identifying histone lactylation, that H3K18 can be both acetylated and lactylated, with competitive occupancy of the two modifications on the same lysine residue determining distinct transcriptional outputs [7]. In peripheral blood mononuclear cells from sepsis patients, Di et al. (2025) further confirmed the clinical significance of this competitive relationship. Critically ill infected patients exhibited significantly elevated H3K18la levels and reduced H3K18ac levels, and the H3K18la/ac ratio demonstrated superior diagnostic performance in identifying infection and assessing disease severity. H3K18la and H3K18ac were associated with distinct inflammatory cytokine profiles. H3K18la positively correlated with the anti-inflammatory cytokine IL-10 and negatively with IL-5, whereas H3K18ac negatively correlated with pro-inflammatory cytokines, including IL-6, IL-1β, and IL-8. This suggests that competitive switching between the two modifications at the same lysine site may mediate the polarization direction of inflammatory responses [134].

At the enzymatic catalysis level, lactylation and acetylation share p300/CBP as core output enzyme systems. Zhang et al. (2019) confirmed in a cell-free system that p300 can utilize lactyl-CoA as a donor to catalyze histone lactylation [7]. A systematic review by Jiang et al. (2026) further indicated that the catalytic pocket of p300 can be competitively occupied by acetyl-CoA and lactyl-CoA, and the stoichiometric ratio of the two acyl-CoAs within the cell directly determines the catalytic output direction of p300 [135]. Binding of both acetyl-CoA and lactyl-CoA to p300 is entropy-driven, but the acetyl group contributes greater hydrophobic interactions, resulting in acetylation predominating under metabolic steady-state conditions [136]. However, under conditions of massive lactate accumulation driven by the Warburg effect, the elevated lactyl-CoA concentration is sufficient to gain an advantage in competitively occupying the p300 catalytic site, thereby switching p300 function from an acetyltransferase to a lactyltransferase [135]. To further illustrate the fine regulation of this competitive modification switching, Zhang et al. (2026) found that NAA50, as an N-acetyltransferase, can inhibit lactylation at the same lysine residue by promoting acetylation at HNRNPU K181, with the two modifications exhibiting a dynamic temporal relationship of reciprocal fluctuation. This finding reveals that competition between acetylation and lactylation depends not only on the global concentration of metabolic substrates but is also subject to local regulation by site-specific acetyltransferases [137].

### 6.2. Competition with Succinylation and Palmitoylation

As a shared site for multiple acylation modifications, lysine residues can be competitively occupied by different types of acyl groups, establishing a post-translational modification regulatory logic determined by metabolite abundance ratios. The cross-regulation of succinylation, palmitoylation, and lactylation on GSDMD, the pyroptosis execution protein, provides a representative example for understanding how metabolites influence cell fate through site competition.

GSDMD, as the final executor of pyroptosis, contains Cys191 (human) or Cys192 (murine) located in a disordered loop region of the N-terminal domain. This region is positioned at the pore apex when GSDMD-NT forms β-barrel pore structures, directly exposed to the solvent environment, making it a sensitive target for multiple post-translational modifications. Palmitoylation of this cysteine residue has been confirmed as a key regulatory step for GSDMD membrane translocation and pore assembly. Balasubramanian et al. (2024) demonstrated through acyl-biotin exchange assays and click chemistry labeling that ZDHHC5- and ZDHHC9-mediated palmitoylation of GSDMD Cys192 enhances the binding affinity of GSDMD-NT to phosphatidylinositol phosphates and cardiolipin, promoting its translocation from the cytosol to the plasma and mitochondrial membranes, thereby driving pyroptotic cell death [138]. Inhibition of palmitoylation significantly reduced GSDMD oligomerization levels and LDH release, whereas the C192A mutation completely abolished palmitoylation signals and delayed pyroptosis kinetics. GSDMD Cys191/192 can also be modified by fumarate-mediated succinylation. Humphries et al. (2020) reported that under conditions of tricarboxylic acid cycle intermediate fumarate accumulation, GSDMD Cys191 undergoes succinylation [139]. This modification inhibits GSDMD oligomerization and pore formation through covalent occupancy of the cysteine residue, thereby blocking pyroptosis. This finding directly links fumarate level fluctuations to reversible regulation of GSDMD activity, with succinylation serving as a negative regulatory mechanism for pyroptosis execution [138,140].

Concurrently, Cys191/192 can also undergo lactylation. Zhang et al. (2019), while identifying histone lactylation, also found that the non-histone protein GSDMD can be lactylated. However, although the mass spectrometry analysis in that study covered multiple lysine residues of GSDMD, it did not identify Cys191 as a lactylation site [7]. Du et al. (2024) subsequently confirmed the presence of S-palmitoylation rather than lysine lactylation at GSDMD Cys191 (human). Given that Cys191 is a cysteine residue, its lactylation would refer to S-lactylation (lactate linked to the cysteine sulfhydryl group via a thioester bond). Currently, direct experimental evidence is lacking regarding whether GSDMD Cys191 undergoes S-lactylation and how such modification might participate in pyroptosis regulation [24].

Chen et al. (2026) systematically elaborated on the cross-regulatory relationships among multiple acylation modifications, noting that succinylation, malonylation, and glutarylation introduce negative charges while also increasing side chain volume, exerting more pronounced effects on protein activity than acetylation or lactylation. The two modifications are diametrically opposed in chemical properties and functional outputs [60]. Chen et al. (2026) further pointed out that metabolite abundance ratios constitute the core variable determining modification types, a principle fully reflected at the GSDMD Cys191 site [60].

The competition among palmitoylation, succinylation, and potential lactylation at GSDMD Cys191/192 constitutes a post-translational modification logic node driven by metabolite abundance in pyroptosis regulation. The same amino acid residue, when occupied by acyl groups derived from different metabolites, produces diametrically opposing biological effects: palmitoylation promotes pyroptosis, succinylation inhibits pyroptosis, and the function of lactylation remains to be elucidated. This case reveals how changes in metabolite species and abundance are converted into cell fate determination signals through differential modification at the same chemical site and also provides a reference paradigm for understanding the cross-regulation between lactylation and other acylation modifications on broader protein substrates.

### 6.3. Crosstalk Between Lactylation and Phosphorylation

Lactylation can influence phosphorylation levels by altering the conformation or charge state of substrate proteins, whereas phosphorylation can serve as an upstream signal to regulate lactyltransferase activity or substrate accessibility. The two modifications form bidirectional regulatory loops in the integration of metabolism and signaling.

The ULK1-LDHA-Vps34 signaling axis provides a representative case of lactylation influencing phosphorylation. Jia et al. (2023) found that under nutrient deprivation conditions, the autophagy-initiating kinase ULK1 directly phosphorylates lactate dehydrogenase A at S196 [141]. This phosphorylation enhances LDHA enzymatic activity and promotes lactate production. Lactate subsequently mediates Vps34 K356 and K781 lactylation through the acetyltransferase TIP60. Lactylated Vps34 enhances its interactions with Beclin1, Atg14L, and UVRAG, elevates its lipid kinase activity, and promotes autophagic flux. In this hierarchical regulation, phosphorylation (ULK1 to LDHA) is positioned upstream in the signaling cascade, whereas lactylation (Vps34) functions as the executor of the phosphorylation signal. ULK1 phosphorylation of LDHA S196 occurs in the loop domain adjacent to the histidine site of the NADH proton acceptor, and phosphorylation may enhance enzyme-cofactor binding efficiency through induced conformational changes [141].

Lactylation can also directly regulate the phosphorylation status and transcriptional activity of transcription factors. Deng et al. (2026) found in an atherosclerosis model that disturbed flow shear stress induces lactate accumulation through enhancing endothelial cell glycolysis, which in turn drives FOXO1 lactylation and nuclear translocation. Lactylated FOXO1 promotes monocyte adhesion through transcriptional activation of inflammation-related genes, and AARS1 deficiency can block this process. Under physiological shear stress conditions, the KLF2-CDK2 signaling axis promotes FOXO1 cytoplasmic retention through phosphorylation at S249, forming an antagonistic relationship with lactate-driven FOXO1 nuclear translocation following lactylation. The coordinated regulation of the two modifications on the same protein constitutes a molecular switch for mechano-metabolic signal integration. Deng et al. (2026) further observed that lactate treatment does not affect FOXO1 S249 phosphorylation levels, and CDK2 deficiency does not affect FOXO1 lactylation, suggesting that phosphorylation and lactylation regulate FOXO1 through independent parallel pathways, responding respectively to physiological shear stress and metabolic stimuli [142].

At the level of lactylation regulating kinase activity, Lan et al. (2026) found in osteoarthritis research that UGDH K6 lactylation not only inhibits its catalytic activity as a dehydrogenase but also alters its nucleocytoplasmic distribution. Under IL-1β stimulation, lactylated UGDH translocates from the nucleus to the cytoplasm, accompanied by a weakened interaction with the transcription factor STAT1. In the nucleus, UGDH binding to STAT1 inhibits STAT1-mediated transcriptional activation of the MAP3K8 promoter, whereas their dissociation resulting from UGDH lactylation promotes MAP3K8 expression and activation of the downstream p38-MAPK signaling pathway. This finding reveals how lactylation indirectly regulates the transcriptional output of phosphorylation signaling pathways by altering protein–protein interaction networks, rather than directly modifying the kinase itself. In a collagen-induced arthritis model, the P300 inhibitor A485 suppressed UGDH K6 lactylation, restored UGDH-STAT1 interaction, and inhibited MAPK signaling pathway activation, providing proof of concept for intervention strategies targeting lactylation-phosphorylation cross-regulation [143].

Phosphorylation can also regulate the direction of lactylation. Hypoxia-induced AARS2 regulation provides an important example for understanding how phosphorylation affects the lactylation enzymatic machinery. Mao et al. (2024) found that under hypoxic conditions, the mitochondrial alanyl-tRNA synthetase AARS2, functioning as a lactyltransferase, catalyzes lactylation at PDHA1 K336 and CPT2 K457/458, thereby inhibiting pyruvate dehydrogenase complex and carnitine palmitoyltransferase 2 activity and limiting oxidative phosphorylation supply. In this process, hypoxia signals are sensed through PHD2 hydroxylation activity. PHD2 hydroxylation of AARS2 P377 promotes its binding to VHL and ubiquitination-mediated degradation. PHD2 itself, as a dioxygenase, depends on oxygen, alpha-ketoglutarate, and ferrous ions for its activity, and its catalytic state is regulated by cellular redox status. Reactive oxygen species generated by oxidative phosphorylation can indirectly regulate AARS2 stability by affecting PHD2 activity. This progressively layered regulatory chain reveals a multi-tiered post-translational modification cascade spanning phosphorylation (PHD2 activity is regulated by phosphorylation modifications), hydroxylation (PHD2 covalent modification of AARS2), and lactylation (AARS2 modification of PDHA1/CPT2). In this system, phosphorylation indirectly regulates the protein stability of the lactylating enzyme by affecting upstream signaling proteins, although direct evidence requires further clarification [30].

Thus, the crosstalk between lactylation and phosphorylation is bidirectional. Phosphorylation can serve as an upstream signal for lactylation, providing substrates for lactylation. Conversely, lactylation can influence phosphorylation signals by altering substrate protein conformation or interaction networks. The two modifications often form hierarchical regulatory networks within the same signaling pathway, with phosphorylation tending to transmit rapid and reversible signal responses, whereas lactylation converts metabolic states into more durable protein functional remodeling. Their complementarity in temporal scales constitutes a fine regulatory mechanism by which cells respond to metabolic fluctuations.

## 7. Future Perspectives and Open Questions

### 7.1. Unresolved Mechanistic Questions

Although lactylation research has expanded from conceptual discovery to multi-level functional validation within just a few years, several core mechanistic questions remain insufficiently addressed. Clarifying these questions will determine whether the field can progress from phenomenological description to causal explanation.

The first aspect is the absence of site specificity and the lactylation code. The occurrence of lactylation on histones and non-histone proteins is not random, yet the molecular logic determining whether a given lysine residue is occupied by lactylation rather than other acylation modifications remains unclear. Existing evidence indicates that the catalytic pocket of p300/CBP can be competitively occupied by acetyl-CoA and lactyl-CoA, and the stoichiometric ratio of the two acyl-CoAs within the cell is an important factor determining catalytic output direction [144]. However, the differential modification of distinct sites by the same p300 molecule within the same nucleus is not solely determined by metabolite concentration. Enzyme activity, the presence of cofactors, and other competitive factors may also contribute to this specificity. Qu et al. (2023) noted that the enrichment of lactylation at transcriptionally active regions, such as H3K18la, positively correlates with gene expression levels, suggesting that chromatin context or pre-existing histone modification marks may provide targeting signals for lactylation [145]. The work of Tsusaka et al. (2025) further revealed that HDAC1-3-catalyzed lactylation may be the primary source of intracellular lactylation, and this process is directly driven by lactate concentration rather than relying on the lactyl-CoA pathway. This finding shifts the underlying logic of the lactylation code from enzymatic preference toward integrated regulation of metabolite concentration and enzymatic activity directionality. However, this study also sparked controversy regarding the specificity of commercial H3K18la antibodies [146]. Furthermore, the molecular basis of substrate selectivity for AARS1/2, which function as lactyl-CoA-independent lactyltransferases, remains unclear. Currently, lactylation lacks clear recognition rules analogous to the kinase-substrate motif in phosphorylation or the bromodomain-acetylated lysine in acetylation.

The second aspect is the subcellular localization of lactyl-CoA and substrate accessibility. The intracellular concentration of lactyl-CoA is extremely low, approximately 1/350 to 1/20 of acetyl-CoA [15], raising questions about its feasibility as a universal acyl donor. Tsusaka et al. (2025) were unable to detect significant elevation of lactyl-CoA levels following lactate treatment in HEK293T cells, further challenging the dominance of the lactyl-CoA-dependent pathway in global lactylation [146]. Although ACSS2 and GTPSCS have been identified as lactyl-CoA synthetases, their substrate supply efficiency and spatial regulatory mechanisms within the nucleus remain unclear. If lactylation predominantly relies on HDAC-catalyzed direct condensation reactions, the subcellular distribution of lactyl-CoA may need to be redefined; it may only function as a primary acyl donor in specific cell types or pathological states. Additionally, whether lactylation can be enriched in particular subcellular compartments such as mitochondria, and how lactylation modifications across different compartments coordinate with each other, currently lack systematic investigation. Mao et al. (2024) identified AARS2-mediated PDHA1 and CPT2 lactylation in mitochondria, suggesting the existence of an independent lactylation regulatory system in mitochondria, but the relationship between the lactyl-CoA supply pathway in this system and the cytosolic pathway remains to be elucidated [30].

The third aspect is the function of lactylation in cancer stem cells and metabolic heterogeneity. Cancer stem cells (CSCs) are highly resistant to conventional therapies and are characterized by enhanced glycolysis and lactate production. A bibliometric analysis by Li et al. (2025) showed that stem cells have emerged as a keyword cluster in lactylation research, but direct mechanistic evidence remains limited [147]. Pan et al. (2022) found that the triterpenoid compound demethylzeylasteral can attenuate the tumorigenicity of liver cancer stem cells by inhibiting H3K9la and H3K56la, providing pharmacological clues for the role of lactylation in CSC regulation. However, whether lactylation drives tumor recurrence and metastasis by maintaining CSC stemness properties, promoting symmetric division, or resisting differentiation requires systematic investigation. Related to this is the issue of tumor metabolic heterogeneity; significant lactate concentration gradients exist across different tumor subtypes and even within different regions of the same tumor, which inevitably leads to spatial heterogeneity of the lactylation modification landscape [148]. Galle et al. (2022) showed that H3K18la can mark tissue-specific active enhancers, but its distribution patterns across different tumor microenvironment regions have not been revealed by spatially resolved omics technologies. The application of single-cell and spatial omics technologies holds promise for filling this gap, but related research remains in its early stages [34].

### 7.2. Technological Aspects

The advancement of lactylation research depends considerably on progress in detection and analytical technologies. Current technical bottlenecks center on sensitive detection of low-abundance modification sites, development of highly specific antibodies, and resolution of modification heterogeneity at the single-cell level. Addressing these issues will provide critical tools for understanding the biological functions of lactylation.

A key bottleneck in mass spectrometry-based lactylation site identification lies in detection confidence. Wan et al. (2022) provided a breakthrough method for high-confidence lactylation identification. This study discovered a cyclic iminium ion formed during the fragmentation of lactylated lysine residues in tandem mass spectrometry, with a characteristic mass-to-charge ratio of *m*/*z* 156.1025 [149]. Large-scale informatics evaluation against non-lactylated spectral libraries confirmed that the false positive interference rate of this diagnostic ion was less than 2.5%, with specificity and sensitivity superior to traditional linear iminium ions. Using this diagnostic ion strategy, the research team successfully identified 137 lactylated peptides from the non-enriched human proteome database (Meltome Atlas), corresponding to 125 modification sites on 83 proteins. More importantly, through genetic code expansion technology to site-specifically introduce lactylated lysine into recombinant aldolase A (ALDOA), this study functionally confirmed for the first time that K147 lactylation inhibits enzymatic activity, revealing the possibility of negative feedback regulation of glycolytic flux through lactylation. This finding also suggests that a substantial amount of unrecognized lactylation information is embedded within existing public proteome databases, and systematic reanalysis will provide a data foundation for elucidating the distribution patterns of this modification.

Another critical bottleneck in mass spectrometry is the detection sensitivity for low-abundance modifications. Traditional data-dependent acquisition (DDA) methods only perform secondary fragmentation on the most abundant precursor ions, leading to systematic neglect of low-abundance lactylated peptides. Data-independent acquisition (DIA), through predefined isolation windows for the fragmentation of all precursor ions combined with targeted extraction from spectral libraries, significantly improves detection reproducibility for low-abundance peptides [150]. Wang et al. (2024) systematically optimized diaPASEF parameters on the timsTOF SCP platform, including a 140 ms ion accumulation time, a 20 *m*/*z* isolation window width, and 60-min chromatographic gradients [43]. Combined with smaller inner diameter columns (50 μm × 10 cm) and the addition of 0.02% DDM surfactant, the number of identified proteins at single-cell equivalent levels (1 ng HeLa peptide) increased 19-fold to 3868 proteins. This methodological advancement demonstrates that through refined instrumental parameter optimization and sample preparation workflow improvements, the detection sensitivity for lactylation modifications can be enhanced by orders of magnitude.

Furthermore, single-cell-level protein analysis has opened a new dimension for resolving the impact of cellular heterogeneity on lactylation modifications within the tumor microenvironment. However, compared to conventional single-cell proteomics, the stoichiometry of lactylation modifications is typically low. At ALDOA K147, the ratio of modified to unmodified peptides across different cell lines ranges from approximately 0.002 to 0.14. This means that in a single cell, the ion signal of lactylated peptides may fall below the detection limit of mass spectrometry. The work of Wan et al. (2022) showed that even in non-enriched proteome data, lactylation sites can be identified on abundant metabolic enzymes using cyclic iminium ion diagnostic signals [149]. For the lactylation of low-abundance signaling proteins or transcription factors, antibody enrichment strategies remain an indispensable pre-processing step. A systematic review by Hu et al. (2025) further pointed out the limitations of lactylation-specific antibodies. Currently available commercial pan-lactylation antibodies, while capable of recognizing lactylated lysine residues, exhibit cross-reactivity with short-chain acylation modifications, including acetylation and propionylation. The development of site-specific antibodies is constrained by the immunogenicity of modified peptides and the difficulty of affinity screening [151].

From the data resource perspective, the establishment of the SPDB database provides infrastructure for systematic integration and analysis of single-cell proteome data [152]. This database includes 143 single-cell proteome datasets covering antibody methods and mass spectrometry, involving over 300 million cells and more than 8000 proteins. However, only 10 single-cell proteome datasets by mass spectrometry are included in the database, involving over 4000 cells and approximately 7000 proteins, far fewer than the scale of datasets from antibody methods. This reflects that the former remains in the early stages of development in terms of throughput and coverage depth. Jing et al. (2025) noted in their review that lactylation site prediction models assisted by artificial intelligence, such as Auto-KLA and the KLA model, have demonstrated preliminary high-throughput screening potential. However, the predictive accuracy of these models depends on the quality and scale of training data, and the currently known number of lactylation sites is insufficient to support adequate training of deep learning models [153].

In summary, lactylation modification technology is at a critical stage, transitioning from qualitative identification to precise quantification and single-cell resolution. The establishment of mass spectrometry diagnostic ion strategies has resolved the question of low-abundance modifications. Optimization of DIA and diaPASEF methodologies has improved detection sensitivity and throughput. The maturation of single-cell proteomics technologies has made it possible to resolve cellular heterogeneity within the tumor microenvironment. However, to truly apply these technologies to systematic functional studies of lactylation, further breakthroughs are still needed in the development of highly specific antibodies, improvement of single-cell lactylomics throughput, and establishment of standardized data analysis protocols.

### 7.3. Clinical Aspects

The multiple regulatory functions of lactylation in tumor development and immune evasion have provided a solid theoretical foundation for its clinical translation. From biomarker development to exploration of targeted therapeutic strategies, lactylation-related pathways are moving from basic research toward clinical application. However, this translational process still faces multiple challenges in selectivity, safety, and efficacy.

In the biomarker field, lactylation modification levels have demonstrated potential as indicators for tumor diagnosis and prognosis assessment. Multiple studies have confirmed that global lactylation and specific histone site modifications such as H3K18la and H4K12la are significantly elevated in tumor tissues compared to adjacent normal tissues, and their elevation correlates closely with shortened overall survival and disease-free survival [151,153]. Serum lactate dehydrogenase levels, as a routine clinical laboratory parameter, have been established as independent prognostic factors for immune checkpoint blockade (ICB) therapy outcomes in multiple retrospective cohort studies. In the reanalysis of the IMpower133 study, extensive-stage small cell lung cancer patients with high serum LDH levels did not derive survival benefit from atezolizumab combined with chemotherapy, whereas patients with low LDH levels had significantly prolonged median survival. Notably, in this cohort, tumor cell PD-L1 expression levels failed to effectively predict immunotherapy response, highlighting the unique predictive value of lactate metabolism-related biomarkers in specific tumor types. At the mechanistic level, LDH levels negatively correlate with the extent of CD8^+^ T cell infiltration and TCR clonal diversity in the tumor microenvironment. The mechanism involves lactate upregulating Nur77 expression in naïve CD8^+^ T cells through H3K18la lactylation, inducing tonic TCR signaling and impairing T cell responsiveness to antigenic stimulation [130]. This finding directly links serum LDH, a routine clinical parameter, to lactylation-driven immune evasion mechanisms, providing an actionable theoretical basis for patient selection in ICB therapy.

Drug development targeting the lactylation enzymatic machinery represents the most challenging direction in clinical translation. The writers of lactylation primarily include acetyltransferase families such as p300/CBP, as well as the recently discovered AARS1/AARS2. However, p300/CBP functions simultaneously as a lactyltransferase and acetyltransferase, and its small molecule inhibitors, including C646 and A-485, inevitably affect the acetylation modification network while blocking lactylation. This broad-spectrum inhibition mode may produce unintended biological effects. The discovery of AARS1/2 as novel lactate sensors and lactyltransferases provides new targets for developing more selective intervention strategies. Zong et al. (2024) confirmed that AARS1 can directly catalyze p53 K120 and K139 lactylation using lactate and ATP, without requiring lactyl-CoA as an intermediate donor [132]. This discovery opens a regulatory channel independent of p300/CBP, but the development of AARS1/2-selective inhibitors remains in early exploratory stages, and clinical validation will require time. At the eraser level, HDAC inhibitors including vorinostat and entinostat can enhance delactylase activity, but they also affect acetylation modifications. HDAC family members exhibit isoform-specific functions. The HDAC6-selective inhibitor Nexturastat A demonstrated synergistic antitumor effects when combined with the anti-PD-1 antibody in melanoma models, with mechanisms involving M1/M2 polarization remodeling of tumor-associated macrophages and increased CD8^+^ T cell infiltration [125]. This finding suggests that isoform-selective HDAC inhibitors may achieve regulation of lactylation without completely sacrificing acetylation modification functions.

Timing and dose optimization of combination therapy represent another critical issue in clinical translation. Preclinical studies have shown that LDHA inhibitors (FX11, GSK2837808A) combined with anti-PD-1/PD-L1 antibodies demonstrate synergistic antitumor effects in non-small cell lung cancer, melanoma, and pancreatic cancer models. The MCT1-selective inhibitor AZD3965 is currently the most clinically advanced lactate transport-targeting drug, having completed Phase I/II clinical trials in advanced solid tumors and diffuse large B-cell lymphoma (NCT01791595), establishing the tolerated dose and pharmacokinetic profile, with dose-limiting toxicity primarily consisting of reversible ocular changes [131]. However, these early clinical studies have predominantly focused on safety and tolerability as primary endpoints and have not systematically evaluated the remodeling effects of these interventions on lactylation modification levels and the immune microenvironment in tumor tissues. Notably, lactate inhibits PD-L1 ubiquitin-proteasome degradation through AARS1-mediated PD-L1 K280 lactylation, thereby stabilizing PD-L1 protein levels. The lactylation-maintained high PD-L1 expression may paradoxically provide more abundant targets for anti-PD-L1 antibodies. This finding reveals a seemingly counterintuitive yet highly translationally valuable strategy: maintaining rather than eliminating PD-L1 expression may optimize ICB efficacy by increasing antibody accessibility. This study validated the association between PD-L1 lactylation and patient prognosis in non-small cell lung cancer tissue microarrays through multiplex immunohistochemistry, providing proof of concept for the feasibility of lactylation as a companion diagnostic biomarker.

From the clinical translation perspective, the functional divergence of lactylation across different cell types constitutes the core challenge in defining the therapeutic window. In tumor cells, lactylation drives malignant progression and immune evasion. In T cells, appropriate lactate levels maintain stem-like phenotypes and enhance antitumor function through lactylation. Systemic suppression of lactate production or lactylation may simultaneously weaken antitumor immune responses. This double-edged sword effect requires combination therapeutic regimens to achieve precise regulation in both spatial dimensions (tumor tissue versus immune organs) and temporal dimensions (pre-treatment versus on-treatment). Currently, no clinical trial has specifically used lactylation modification levels as patient enrollment criteria or efficacy predictive indicators. The prognostic predictive value of lactylation-related gene signatures is mostly based on construction and validation from retrospective public databases, and their generalizability across different populations and treatment contexts requires prospective cohort validation. Furthermore, the dynamic reversibility of lactylation implies that static tissue biopsy biomarkers may not fully capture real-time changes in the lactylation landscape during treatment. Integrating lactylation modificomics monitoring in liquid biopsy with imaging assessment may represent a direction for optimizing combination therapeutic strategies in the next phase.

Clinical translation of lactylation modification is at a critical juncture, transitioning from proof of concept to targeted intervention. Future research should focus on establishing patient stratification methods based on lactylation modification profiles, systematic evaluation of optimal combination therapy timing and dosing, and prospective validation incorporating lactylation modification levels into clinical trial designs.

## 8. Conclusions

The discovery of lactylation modification has fundamentally reshaped the biological connotation of lactate, a traditional metabolite. Once regarded as a terminal waste product of glycolysis, lactate now reveals its capacity to covalently modify lysine residues, directly coupling cellular metabolic states to protein functional regulation and gene expression programs. This establishes a direct molecular bridge from metabolic flux to phenotypic output. Understanding the functional logic of lactylation requires systematic appreciation of the multi-source supply characteristics of lactate, the generation pathways of lactyl-CoA, the discovery of novel lactyltransferases, the substrate selectivity of delactylase families, the functional divergence between histone and non-histone substrates, and the competitive interplay between lactylation and other acylation modifications. In the context of tumor biology, lactylation operates through two regulatory dimensions. Within tumor cells, it drives sustained activation of proliferative signals, positive feedback reprogramming of metabolic networks, evasion of cell death programs, and acquisition of invasive and metastatic capacities through histone epigenetic remodeling and non-histone protein functional modulation. Within the tumor microenvironment, it constructs a vicious cycle between metabolism and immunosuppression by reprogramming the polarization direction of tumor-associated macrophages, inducing functional exhaustion of CD8^+^ T cells, and stabilizing immune checkpoint protein expression. This dual regulatory mode of cell-autonomous and microenvironment non-autonomous actions positions lactylation as a critical driver node in malignant tumor progression and therapeutic resistance.

Intervention strategies targeting the lactate metabolic pathway or lactylation enzymatic machinery have shown synergistic potential with immune checkpoint inhibitors in preclinical models. A limited number of agents, including LDH inhibitors, MCT inhibitors, and HDAC modulators, have progressed to early-stage clinical trials, though largely for safety and tolerability assessment rather than efficacy validation. However, clinical translation of lactylation modification still faces fundamental challenges. The shared enzymatic regulatory network between lactylation and acetylation presents structural pharmacology difficulties for selective intervention. The diametrically opposing functional outputs of lactylation in tumor cells versus effector immune cells demand precise definition of the therapeutic window. The dynamic reversibility and tissue heterogeneity of the modification impose new spatiotemporal requirements on biomarker development. Future research should focus on spatiotemporal dynamic resolution of lactylation and elucidation of site-specific regulatory mechanisms, development of patient stratification strategies based on modification profiles, and systematic assessment of the optimal timing and dosing combinations of targeted lactylation combined with immunotherapy through prospective clinical trials. Only by establishing closer dialogue between basic mechanistic investigation and clinical translational research can lactylation, as a nexus of metabolic and epigenetic cross-regulation, truly transition from experimental discovery to a new cornerstone of precision cancer therapy.

## Figures and Tables

**Table 1:** Key regulatory enzymes and substrates of lactylation.

Category	Enzyme	Function	Example Substrates	Ref.
Lactyl-CoA synthetase	ACSS2, KAT2A	ACSS2 catalyzes the conversion of lactic acid to lactyl-CoA; KAT2A utilizes lactyl-CoA to mediate histone H3 lactylation	Histone H3	[17]
	GTPSCS	Directly catalyzes the production of lactyl-CoA and synergizes with p300 to drive histone lactylation	Histone H3K18	[18]
Lactyltransferase	p300/CBP	Catalyzes the transfer of lactyl group from lactyl-CoA to lysine residues	Histones, HMGB1	[16,19]
	HBO1 (KAT7)	Directly catalyzes histone H3K9 lactylation, positively regulated by JADE1/BRPF2	Histone H3K9	[20]
	KAT8 (MOF)	Catalyzes site-specific lactylation, enhances the activity of substrate GTPase and promotes protein synthesis	eEF1A2 K408	[16]
	AARS1/2	Preferentially catalyzes lactylation of non-histone substrates	p53 K120/K139, PDHA1, CPT2	[21,22]
	ATAT1, HDAC6	Exhibit lactyltransferase activity under specific conditions	—	[23]
Delactylase	HDAC1-3	Broad-spectrum removal of lactyl groups to maintain global lactylation homeostasis; HDAC3 possesses the strongest activity, while HDAC1/2 prefers D-lactylation	p53 K382, NF-κB p65, α-tubulin	[8]
	SIRT1	NAD^+^-dependent site-specific delactylation regulates RNA metabolism-related proteins	PKM2 K207	[24]
	SIRT2	Site-specific delactylation regulates cuproptosis	METTL16	[25]
	SIRT3	NAD^+^-dependent site-specific delactylation regulates chromosome localization and DNA damage response	H4K16la, H3K9la, CCNE2 K348la	[26,27,28]

**Table 2:** Representative functions of lactylation in core cancer hallmarks.

Cancer Hallmark	Substrate/Target	Modification Site	Functional Effect	Ref.
Proliferation & Survival	PKM2	K62	Restricts glycolysis via negative feedback in inflammatory macrophages.	[43]
	HMGB1	Lysine residues	Drives exosomal HMGB1 release and increases vascular permeability.	[19]
	METTL3 promoter (Histone H3)	H3K18la	Suppresses CD8^+^ T cell proliferation and cytotoxic function.	[44]
Metabolic Reprogramming	Histones H3/H4 (at glycolytic genes, e.g., HK1, PKM)	H3K18la, H4K8la, etc.	Forms a positive feedback loop that reinforces glycolytic gene transcription.	[45]
	CPT1A (promoter & protein)	H3K18la (promoter); K180/K285 (protein)	Amplifies fatty acid oxidation via transcriptional activation and protein stabilization.	[42]
	NEDD4 promoter/HK2 promoter	H3K14la; H3K18la	Reinforces glycolysis and mediates chemotherapy resistance.	[46]
	TOP2A/NCAPD3 pathway	Indirect (histone lactylation)	Forms a closed loop that sustains glycolysis and tumor growth.	[47,48]
	PDHA1, CPT2	K336 (PDHA1); K457/K458 (CPT2)	Suppresses mitochondrial oxidative phosphorylation under hypoxia.	[30]
	HIF-1α	K12 (human/pig); K644 (mouse)	Stabilizes HIF-1α, upregulating glycolysis and suppressing OXPHOS.	[39]
	GRAMD1A promoter; ACLY enhancer; HOXB13 promoter	H3K9la; H3K18la	Drives cholesterol synthesis, fatty acid synthesis, and lipid accumulation.	[49,50,51]
	SLC6A14 promoter; Glutaminase	H3K18la	Enhances glutamine uptake and anaplerosis.	[52]
	Fis1	K20	Promotes excessive mitochondrial fission and ATP depletion.	[40]
Cell Death	RUBCNL promoter; CNN1 promoter	H3K18la; H4K12la	Promotes autophagic flux and mediates drug resistance.	[53,54]
	NOD2 promoter; PKM2	H3K18la; K433	Drives pyroptosis; regulates PKM2 nuclear translocation via lactylation/acetylation competition.	[55,56]
	ATF3/ATF4/CHAC1 promoters	H3K18la (reduced)	Suppresses ferroptosis by reducing H3K18la at pro-ferroptotic gene promoters.	[57]
	p53; SUCLG2/DLAT axis	K120/K139; H4K16la	Inhibits p53-mediated apoptosis; promotes apoptosis through lactylation-dependent metabolic reprogramming.	[58,59]
Invasion & Metastasis	HAS2 promoter	H3K18la	Activates HAS2, driving EMT and immune evasion via c-MYC/PD-L1.	[60]
	Snail1	Lysine residues	Promotes Snail1 nuclear translocation and represses E-cadherin, driving EMT.	[61]
	YY1	K183	Promotes p53 degradation via FBXO33, relieving EMT suppression.	[62]
	L1CAM/SLIT1 promoters1f	H3K18la	Activates perineural invasion genes in pancreatic cancer driven by CAF-derived lactate.	[63]

**Table 3:** Inhibitors targeting lactate metabolism to suppress lactylation.

Target	Inhibitors	Mechanism	Tumor Model Effects	Clinical Stage	Ref.
LDH (LDHA)	FX11	Inhibits LDHA reduces lactate and lactylation substrate	NSCLC, pancreatic cancer synergizes with anti-PD-1	Preclinical	[97,99,100]
	GSK2837808A	Inhibits LDHA.	Melanoma enhances ACT	Preclinical	[95,101]
	Stiripentol	Inhibits LDHA, reduces H3K9 lactylation	Glioblastoma sensitizes temozolomide	Preclinical (approved for epilepsy)	[98]
	PDT-BIPA	Inhibits LDHA shifts to OXPHOS induces mitochondrial apoptosis	Leukemia TGI > 60% at 1.6 mg/kg	Preclinical	[102]
MCT1/4	AZD3965	Selective MCT1 inhibition, blocks lactate uptake	Solid tumors, DLBCL synergizes with anti-PD-1	Phase I/II (NCT01791595)	[99]
	VB124	Inhibits MCT4, blocks lactate efflux	Hepatocellular carcinoma synergizes with anti-PD-1	Preclinical	[103]
	7-aminocoumarin derivatives	Dual inhibition of MCT1/4 avoids compensatory transport	Blocks lactate transport (models not specified)	Preclinical	[14]
GLUT1/5	BAY-876	Selective GLUT1 inhibition limits glucose uptake	Multiple solid tumors Antiproliferative	Preclinical	[104]
	MSNBA	Inhibits GLUT5 (fructose transporter)	Colon cancer Antiproliferative	Preclinical	[105]
PDHK1	DCA	Inhibits PDK1, activates PDH, promotes pyruvate entry into TCA	NSCLC synergizes with EGFR-TKIs/radiotherapy sensitizes CAR-T	Preclinical (repurposed)	[106]
	compound 1f	Potent PDK1 inhibitor	4T1 breast cancer nano-delivery raises TGI from 35% to 90%	Preclinical	[107]
LDHA + PDK/TCA	CPI-Z2	Dual blockade: LDHA knockdown PDHC/KGDHC inhibition	Triple-negative breast cancer single-injection TGI 90% reverses hypoxia/acidosis promotes T-cell infiltration	Preclinical	[108]

## Data Availability

No new data were created or analyzed in this study. Data sharing is not applicable to this article.
